# Development and validation of a multi-analyte GC-MS method for the determination of 84 substances from plastic food contact materials

**DOI:** 10.1007/s00216-020-02758-7

**Published:** 2020-06-24

**Authors:** Emmanouil D. Tsochatzis, Joao Alberto Lopes, Eddo Hoekstra, Hendrik Emons

**Affiliations:** 1 European Commission, Joint Research Centre (JRC), Retieseweg 111, 2440 Geel, Belgium; 2 European Commission, Joint Research Centre (JRC), Via E. Fermi 2749, 21027 Ispra, Italy

**Keywords:** Food contact materials, Migration into food simulants, Multi-analyte method, GC-MS, Substances migrating from plastic food packaging

## Abstract

Chemical substances shall not migrate from food contact materials (FCM) at levels that are potentially harmful for the consumers. Each of the current analytical methods applied to verify the migration of substances from FCM covers only one or few substances. There is a very limited number of publications on the development of analytical methods allowing the simultaneous determination of several classes of FCM substances, and almost none of them reported methods entirely dedicated to the ones in the positive list of Commission Regulation (EU) No. 10/2011 for plastic FCMs. Therefore, a simple, sensitive and reliable multi-analyte method was developed for the analysis of FCM substances in food simulants. It employs an optimised liquid-liquid extraction with dichloromethane as extraction solvent in the presence of 10% m/v NaCl, followed by quantitative analysis with gas chromatography coupled to mass spectrometry (GC-MS). A combination of total ion chromatograms (TICs) and extracted ion chromatograms (EICs) was used. The optimisation and validation of the method have been carried out according to current international guidelines. Adequate sensitivity was demonstrated in the selected concentration ranges for most of the analytes, with limits of quantification (LOQs) at least three times lower than the legislative limit, when existing. The results showed that the method is sufficiently accurate for the majority of substances, with recoveries between 70 and 115% and relative standard deviations (RSDs) smaller than 20% at three concentration levels. The method was applied to the analysis of some FCM multilayers. The method allows, for the first time, the simultaneous quantification of 84 FCM substances in two of the official food simulants (A and C) at levels of a few ng g^−1^.

**Electronic supplementary material:**

The online version of this article (10.1007/s00216-020-02758-7) contains supplementary material, which is available to authorized users.

**Figure:**
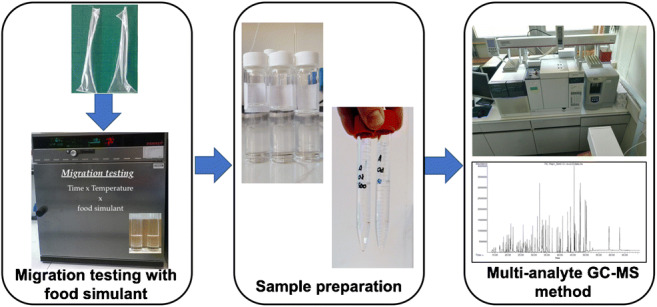
Graphical abstract

## Introduction

Human exposure to chemicals from food contact materials (FCM) occurs mainly as a result of migration from materials into foodstuffs. The extent of this migration is one of the key factors for the human health risk which a packaging material could represent [1].

The European Union (EU) has set up with Regulation (EU) No. 10/2011 a positive list of substances that are allowed to be used in plastic FCM [2]. These substances may have specific migration limits (SMLs) or other restrictions for their application. Another class of frequently found but not regulated substances in FCM is the so-called non-intentionally added substances (NIAS). These are by-products from production processes or they originate from the degradation of materials and could migrate into food.

Official food simulants that mimic the use and properties of real food are also described in the Regulation, and must be used by laboratories when performing migration experiments. The European Union Reference Laboratory for Food Contact Materials (EURL-FCM) maintains and updates a repository of approved FCM additives. From almost 900 regulated substances, less than 600 are commercially available from trustworthy suppliers [2]. Official Control Laboratories (OCLs) all over the EU perform the monitoring of substances used in plastic FCM. Few CEN methods are in place, and therefore, OCLs apply in-house validated methods which focus mainly on one or a few substances.

The availability of validated methods for the simultaneous analysis of large groups of regulated substances listed in the Regulation (EU) No. 10/2011 would improve considerably the efficiency of compliance testing in the plastic FCM field. However, the challenges for the development of such methods are considerable. The Regulation includes not only individual organic substances but also many mixtures, natural products, resins, monomers, oxides, silicates and more. Therefore, the development of multi-analyte methods has to take into account a multitude of different chemical structures and physical-chemical properties. Additionally, such methods cannot be only focused on their instrumental separation and quantification steps, but have to include also a robust sample preparation step that can be applied to the official food simulants. Those may vary from ethanolic solutions to vegetable oils, acetic acid solutions and poly(2,6-diphenyl-p-phenylene oxide), a simulant for dry foods. The methods need also to be very versatile in their sensitivity, as existing SMLs can range from 10 μg kg^−1^ to 30 mg kg^−1^ levels. In some cases, the legislation refers to a maximum mass fraction of substance(s) in the FCM that has to be controlled, typically via extraction followed by measurement techniques. In such cases, sample preparation techniques are even more important due to the complexity of the matrices to be investigated.

Only a limited number of papers related with the analysis of multiple analytes in the FCM field are available in the literature, most of them on materials not regulated at EU level (e.g. paper and board). Representative examples are presented in Table 1.

**Table 1:** Examples of methods for the simultaneous analysis of several substances from FCMs

Type of target analytes	Purpose of the substance in the FCMs	No. of target analytes	Matrix	Analytical technique	Sample preparation	Ref.
Acrylates	Adhesives	7	Food contact paper	GC-MS	QuEChERS	[3]
Benzoxazolyl-based substances (different types)	Fluorescent whitening agents (FWAs)	7	Polystyrene (PS) and polyvinylchloride (PVC) food packaging	UPLC-MS/MS	Extraction, dilution, centrifugation	[4]
Benzoxazolyl-based substances and benzophenones	FWAs and photoinitiators (PIs)	14	Food packaging coated paper	UPLC-MS/MS	Extraction, dilution, centrifugation	[5]
Benzoxazolyl-based substances	FWAs	6	Food packaging cups	HPLC-FLD	Extraction	[6]
Bisphenols, 4-cumylphenol and dihydroxybenzophenone	Monomers, raw materials, contaminants	11	Glass, plastic and multilayers FCMS	GC-MS	Solid phase extraction (SPE) and derivatisation	[7]
Several types	Regulated substance (several functions) and NIAS	14	Plastic baby bottles	GC-MS	Liquid-liquid extraction (LLE), centrifugation, evaporation	[8]
Aromatic amines and benzoxazolyl-based substances (different types)	FWAs and azo dyes (colourants)	13	Food contact paper	HPLC-UV	Subcritical water and dynamic sonication-assisted solvent extraction	[9]
Stilbene derivatives	FWAs	11	Food contact paper and board	HPLC-FLD	Ultrasonication extraction and centrifugation	[10]
Several types	Photoinitiators and amine synergists	63	FCMs and foodstuffs	UPLC-MS/MS	QuEChERS	[11]
Phenol and benzophenones derivatives	Antioxidants, UV absorbers, fire retardants	17	Plastic food packaging extracts	UPLC-PDA	Ultrasonic extraction	[12]
Several groups	Plasticizers, antioxidants, UV absorbers	18	Food packaging	UPLC-MS	Sorptive phase extraction	[13]
Several groups	Dialkylphthalates, bisphenols, printing ink photoinitiators, polyfluoroalkyl substances	41	FCM contaminants in fatty food	UPLC-MS	SPE, LLE, refrigeration	[14]

Most of the target analytes covered by the methods of Table 1 are not regulated in the EU and, therefore, have no legal limits (LLs) in place. The majority of the reported analytical methods aim to detect the presence of analytes in extracts/migration solutions. It also appears that the majority of the target substances were selected either based on a shared chemical nature or on their function as substance in the FCM items. Complex sample preparation steps are often employed, depending on the nature of the article and target substances.

The scope of this study was to develop a method dedicated to the simultaneous quantification of a large group of substances that can be present in plastic FCM. This method should be simple and accurate, as well as being applicable to the analysis of certain official (liquid) food simulants from EU regulation. The need of limited sample preparation was also one of the desired characteristics for the method, together with the possibility of quantification at the legislated migration limits. Its applicability to some real plastic FCM films has been tested.

## Considerations for the development of a dedicated multi-analyte method

The positive list of the Regulation (EU) No. 10/2011 contains more than 900 additives, belonging to different chemical classes/nature and different physical-chemical properties. These substances can range from inorganic to organic, from polar to apolar, from volatile to non-volatile substances, from low molecular masses to masses higher than 1000 Da [2].

**Fig. 1:**
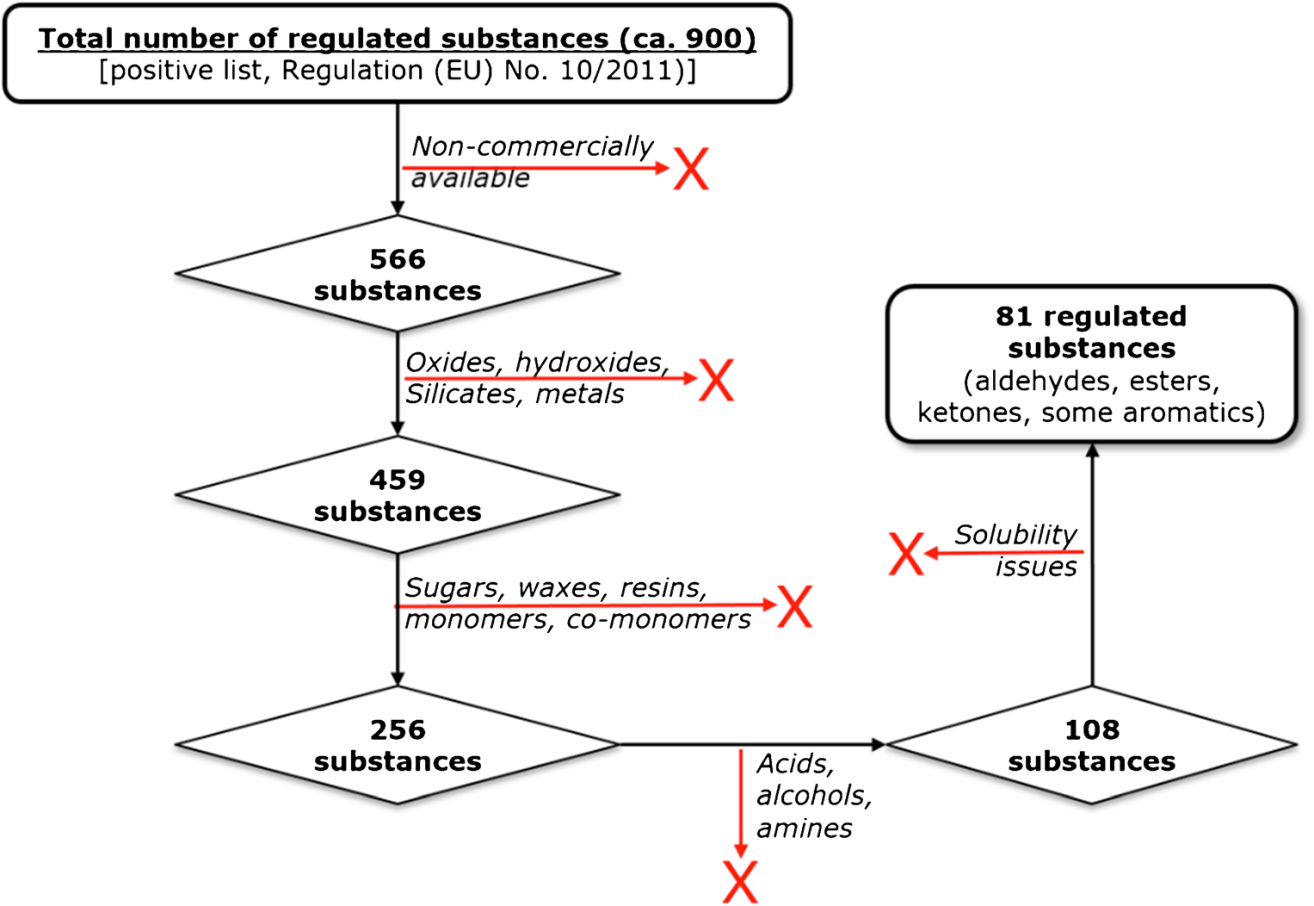
Selection path for the final group of analytes to be targeted by the multi-analyte method

In order to select the group of target analytes for the development of the multi-analyte method, a strategy had to be developed. It has been taken into account for the present study the chemical structure of substances potentially migrating from plastic FCMs, the availability of well characterised analytical standards and a preference for the instrumental approaches intended to be used. A schematic illustration of the process applied to select the final group of analytes to be addressed by the method is presented in Fig. 1.

The first step consisted of removing from the initial number of regulated substances the ones that are not available in the EURL repository for plastic FCM additives. A second step eliminated the substances considered as “inorganic” (oxides, hydroxides, silicates, etc.). Gas chromatography (GC) has been selected due to its versatility and availability in most control laboratories as the analytical separation technique to be used for this method. Therefore, all the non-volatile substances were removed (sugars, waxes, resins, monomers and co-monomers).

The selection of the analytical column is a crucial step for the development of any GC-based method. Here, a 5% phenyl methyl siloxane column has been chosen. This type of stationary phase was not only adequate for the analysis of substances with the chemical nature of most of the selected substances, but allows also a good efficiency and performance at the high oven temperatures expected for the chromatographic programme [15]. Additionally, it was important to select a column offering some resistance towards water as the method should be applied to extracts from aqueous/ethanolic simulants, which may contain traces of water. Therefore, the possibility of an ultra-inert (UI) feature was considered during the column selection. Taking the suitability for using a common 5% phenyl methyl siloxane GC column into account, acids, alcohols and amines were excluded as analytes. Finally, substances which showed a poor solubility during the initial testing in the selected solvents have been excluded. The resulting group covered aldehydes, ketones, esters and some aromatic substances, in total 81 regulated substances as presented in Table 2 (“Chemicals” section). In addition, some NIAS of interest were included as analytical targets, elevating the final number of substances to 84.

**Table 2:** Characteristics of the analysed substances

Analyte	FCM no. *	CAS no.	Purity**	M (Da)	EIC selected ions (*m/z*)***
Hexadecyltrimethylammonium bromide	104	57-09-0	≥ 98%	364.4	58
Camphor	136	76-22-2	≥ 95%	152.2	95
Tri-n-butyl acetyl citrate	138	77-94-1	≥ 97%	402.5	185
Triethyl citrate	140	77-93-0	An. Stand.	276.3	157
Vinyltriethoxysilane	142	78-08-0	97%	190.3	145
4,4′-Dichlorophenyl sulphone	152	80-07-9	98%	287.2	158.9
Dapsone (4,4′-diaminodiphenyl sulphone)	153	80-08-0	An. Stand.	248.3	108
α-Pinene	155	80-56-8	98%	136.2	136
Dibutyl phthalate	157	84-74-2	CRM	278.3	148.9
Benzyl butyl phthalate	159	85-68-7	An. Stand.	312.4	148.9
2,2′-Methylene bis(4-ethyl-6-tert-butylphenol)	163	88-24-4	-	368.2	191.1
Methyl benzoate	171	93-58-3	99%	136.2	105
Ethyl benzoate	172	93-89-0	≥ 99%	150.2	105
Propyl paraben	173	94-13-3	≥ 99%	180.2	121
Allyl methacrylate	175	96-05-9	98%	126.2	57.1
Ethyl methacrylate	181	97-63-2	99%	118.1	69
Isobutyl methacrylate	183	97-86-9	97%	142.2	69
Butyl methacrylate	184	97-88-1	99%	142.2	69
Ethylene dimethacrylate	185	97-90-5	98%	198.2	69
4-tert-Butylphenol	186	98-54-4	99%	150.2	135
α-Methylstyrene	187	98-83-9	99%	118.2	118
methyl paraben	189	99-76-3	≥ 98%	152.2	121
Styrene	193	100-42-5	≥ 99%	104.2	104
Benzaldehyde	195	100-52-7	≥ 99.5%	106.1	106
Cyclohexyl methacrylate	197	101-43-9	≥ 97%	168.2	69/87
Resorcinol diglycidyl ether	199	101-90-6	-	222.2	222
2-Ethylhexyl acrylate	206	103-11-7	98%	184.3	55
Bis(2-ethylhexyl) adipate	207	103-23-1	99%	370.6	129/57.0
2-Ethyl-1-hexanol	209	104-76-7	≥ 99%	130.3	57
Caprolactam	212	105-60-2	99%	113.2	55/113
p-Cresol	216	106-44-5	An. Stand.	108.1	107
1,4-Dichlorobenzene	217	106-46-7	≥ 99%	147	145.9
Isobutyl acrylate	218	106-63-8	≥ 99%	128.7	55
Glycidyl methacrylate	220	106-91-2	97%	142.2	69
Phenol	241	108-95-2	≥ 99.5%	94.1	94
Dibutyl sebacate	242	109-43-3	≥ 97%	314.5	241
Erucamide	271	112-84-5	99%	281.5	59
Bis(2-ethylhexyl) phthalate (DEHP)	283	117-81-7	≥ 99.5%	390.6	148.9
Methyl salicylate	284	119-36-8	≥ 99%	152.2	120
2,2′-Methylene bis(4-methyl-6-tert-butylphenol)	285	119-47-1	-	340.2	177.1
Ethyl paraben	287	120-47-8	99%	166.2	121
Dimethyl terephthalate	288	120-61-6	≥ 99%	194.2	163
Triethylphosphite	293	122-52-1	98%	166.2	83
Butyl acetate	300	123-86-4	≥ 99.5%	116.2	56
Butyl stearate	301	123-95-5	An. Stand	340.6	56
Diphenyl sulphone	313	127-63-9	97%	218.3	124.9
β-Pinene	314	18172-67-3	99%	136.2	136
Butylated hydroxytoluene	315	128-37-0	≥ 99%	220.4	205
Diallyl phthalate	316	131-17-9	An. Stand.	246.3	149
2,4-Dihydroxybenzophenone	318	131-56-6	99%	214.2	137
Butyl benzoate	320	136-60-7	99%	178.2	105
Butyl lactate	322	138-22-7	98%	146.2	57
n-Butyl acrylate	325	141-32-2	≥ 99%	128.2.	55
Oleamide	335	301-02-0	≥ 99.5%	337.6	59
4,4′-Difluorobenzophenonen	337	345-92-6	99%	218.2	122.9
Caprolactone	342	502-44-3	97%	114.1	55
tert-Butyl methacrylate	355	585-07-9	98%	142.2	69
Ethylene glycol monoacrylate	371	818-61-1	96%	116.1	55
Ethylene glycol monomethacrylate	374	868-77-9	≥ 99%	130.1	69
2-Hydroxypropyl acrylate	385	999-61-1	-	130.1	55
1,4-Divinyl benzene	405	1321-74-0	80:20	130.2	130
1,2-Divinyl benzene					
Dimethyl isophthalate	420	1459-93-4	99%	194.2	163
Bisphenol A glycidyl ether (BADGE)	426	1675-54-3	-	340.4	325.1
2-Hydroxy-4-n-octyl benzophenone	431	1843-05-6	98%	326.4	213
Irganox 1076	433	2082-79-3	99%	560.6	530.5
1,4-Butanediol dimethacrylate	434	2082-81-7	95%	226.3	69
Vinyl laurate	436	2146-71-6	≥ 99%	226.4	123
Dodecyl acrylate	437	2156-97-0	90%	240.4	55
Bis (2,6-diisopropylphenyl)-carbodiimide	438	2162-74-5	> 98%	362.6	347.2
Phenyl methacrylate	439	2177-70-0	90%	162.2	69
Propyl benzoate	441	2315-68-6	99%	164.2	105
Benzyl methacrylate	447	2495-37-6	99%	176.2	91
Vinyltrimethoxysilane	453	07/02/2768	98%	148.2	121
sec-Butyl methacrylate	457	2998-18-7	-	142.2	69
1,1,1-Trimethylolpropane trimethacrylate	463	3290-92-4	Techn. grade	338.4	69
Etocrilene	487	5232-99-5	98%	277.3	277
Octocrylene	492	6197-30-4	97%	361.5	249
2,2,4-Trimethyl-1,3-pentanediol diisobutyrate	497	6846-50-0	≥ 98.5%	286.4	71
Irgafos 168	671	31570-04-4	98%	646.9	441.3
[3-(methacryloxy)propyl]trimethoxysilane	788	2530-85-0	≥ 98%	248.4	121
Dioctyl terephthalate	798	6422-86-2	99%	390.6	70
(Z)-Dibutyl maleate	NIAS**	105-76-0	96%	228.3	98.9
Diethyl phosphite	NIAS**	762-04-9	98%	138.1	82
Diisobutyl phthalate (DiBP)	NIAS**	84-69-5	An. Stand.	278.3	149
3-(4-Isopropylphenyl)-2-methylpropionaldehyde	IS 1**	103-95-7	≥ 95%	190.1	133
Diphenyl phthalate	IS 2**	84-62-8	99%	318.3	225

*As stated by the supplier

**IS, internal standard; *NIAS*, non-intentionally added substance

***The most abundant ion (m/z) in the mass spectrum of each substance has been selected for the EIC analysis.

The analytical method was developed for use with official food simulants A and C (10% and 20% v/v aqueous ethanol, respectively). These food simulants tend to be relatively simple matrices, requiring limited sample preparation (extraction/clean-up) steps. However, a “change of solvent” step has to be included for GC methods, as water-containing samples should not be analysed directly. This solvent change is commonly achieved through a simple liquid-liquid extraction (LLE) with a proper organic solvent. The challenge here was to identify an organic solvent that acts as an efficient extraction solvent and can also solubilise the broad range of target substances. The solvents have also to be compatible with GC-MS, i.e. they should not add any analytical interference or shorten the lifetime of the analytical column.

## Materials and methods

### Chemicals

Ethanol (EtOH; CAS: 64-17-5), n-hexane (Hex; CAS: 110-54-3), isooctane (Iso; CAS: 540-84-1), tert-butyl methyl ether (MTBE; CAS: 1634-04-4) and dichloromethane (DCM; 75-09-2) were Chromasolv grade purity and obtained from Sigma-Aldrich (Steinheim, Germany). Ultrapure water (18.2 MΩ), for the preparation of the official food simulants (A and C, 10% and 20% v/v aqueous ethanol, respectively) and solutions, was obtained from a Milli-Q system (Millipore, Bedford, USA). Sodium chloride (NaCl; ≥ 99.5%) was supplied from Fluka (Steinheim, Germany). PTFE 17 mm, 0.2 μm membrane filters were supplied from CPS Analitica (Milan, Italy).

All the analytical standards were obtained either from Sigma-Aldrich (Steinheim, Germany) or from TCI Chemicals (Tokyo, Japan). All the relevant information regarding the target substances are presented in Table 2, including FCM numbers, CAS numbers, molecular masses, purity (as stated by the supplier) and MS data regarding the selected abundant ions (*m/z*) for extraction ion chromatograms (EIC) [2]. In addition, three NIAS were included, i.e. di-n-butyl maleate, diisobutyl phthalate (DiBP) and diethyl phosphite. Tributyl aconitate, a by-product of acetyl tributyl citrate, was identified but it has not been quantified (please see “Experimental issues in the quantification of some FCM substances” section). The DiBP was included because of EFSA’s reassessment of phthalates in FCM [16], while diethyl phosphite originates from the degradation of FCM No. 293. The method can also qualitatively assess the degradation of FCM No. 138 (tri-n-butyl acetyl citrate) into tributyl aconitate.

### Preparation of standard solutions

Stock solutions containing 10 mg mL^−1^ of each analyte and internal standards (IS) were prepared using ethanol as solvent and were stored at − 18 °C. Appropriate working solution mixtures were prepared by diluting the stock standard solutions with ethanol and sonicated (59 kHz) at 25 °C for 15 min. Stock solutions were stored at − 18 °C, while the working solutions were stored at 4 °C. Fresh working standards were produced every week. Both stock and working standard solutions were prepared in amber vials in order to prevent any light-induced degradation or isomeric conversion of the substances. As contamination with phthalates is very common during sample preparation procedures [3, 4, 17, 18], only glassware properly cleaned and rinsed was used. Briefly, all glassware was rinsed twice with acetone and hexane and stored in a desiccator over aluminium oxide [17, 18].

### Analysis of real FCM samples

Fifteen plastic FCM samples coming from a range of EU plastic producers have been tested as to check the applicability of the method to real samples. Samples were stored in wrapped aluminium foils at room temperature (20 ± 5 °C). The test conditions of the migration experiments were based on the intended use of the material according to Regulation (EU) No. 10/2011 [2]. All the samples were cut into square pieces (approximately 1 dm^2^; 10 × 10 cm) prior to the test. Data regarding the type of material, the intended use, the type of food simulant, the type of migration experiment, the amount of food simulant and the contact time and temperature conditions are presented in Table 3.

**Table 3:** Description of the analysed FCM samples, type and volume of food simulants, type of migration test and the specific time-temperature conditions

Sample code	Material type	Type of material	Intended use	Food simulant*	Amount of food simulant (mL)	Migration experiment	Time-temperature conditions
S5	Monolayer	Polypropylene (PP)	Salad pot	A	350	Filling	20 °C × 10 d
S13	Multilayer	Polyamide (PA) /ink	Sausage	A	35	Pouch, 1 dm^2^	40 °C × 10 d
S20	Monolayer	PP film	Vegetables	A	100	Immersion, 1 dm^2^	60 °C × 10 d
S22	Multilayer	PP copolymer	Vegetables, fruits	A	100	Immersion, 1 dm^2^	60 °C × 10 d
S25	Multilayer	PET/PETG/ LLDPE	Hot liquids	C	50	Pouch, 1 dm^2^	60 °C × 10 d
S29	Monolayer	HDPE	Vegetables, fruits	A	100	Immersion, 1 dm^2^	60 °C × 10 d
S31	Monolayer	PP	Vegetables, fruits	A	100	Immersion, 1 dm^2^	20 °C × 10 d
S34	Monolayer	PP	Vegetables, fruits	A	100	Filling	40 °C × 10 d
S41	Monolayer	PVC	Honey	A	100	Immersion, 1 dm^2^	60 °C × 10 d
S44	Monolayer	PVC	Jam	C	100	Immersion, 1 dm^2^	60 °C × 10 d
S49	Multilayer	PA/LLDPE	sausage	A	50	Pouch, 1 dm^2^	60 °C × 10 d
S62	Monolayer	PVC	Processed meat	A	100	Immersion, 1 dm^2^	40 °C × 10 d
S71	Monolayer	PP	Ice cream	C	100	Immersion, 1 dm^2^	20 °C × 10 d

*According to Annex III, Tables 1 and 2 of Regulation (EU) No. 10/2011 [2]

### Liquid-liquid extraction

The used LLE method was based on the sample preparation procedure known as QuEChERS (quick, easy, cheap, effective, rugged and safe) [19] and on previous work carried at the EURL-FCM, with slight modifications [20]. In the present study, a specimen of 5 mL of the food simulant (A or C), containing 400 mg of NaCl, the 2 selected IS and 2 mL of DCM were added to a tube. The tube was vigorously vortexed for 1 min and centrifuged using an Eppendorf 5810 R refrigerated centrifuge, set at 20 °C and 2500 rpm (1280*g*) for 5 min. The procedure was repeated for a second time by adding 1 mL of DCM. The DCM extracts were removed (bottom solvent layers), filtered with PTFE 0.22 μm filters and transferred to another glass tube, where they have been concentrated to 150 μL by a gentle stream of nitrogen at 25 °C, and the volume fixed to 300 μL by adding DCM.

### GC-MS analysis

The method was designed to rely mainly on the separation power of the chromatographic step rather than taking advantage of using an MS detector. That means chromatographic resolution was a crucial factor to be considered. Therefore, a 60-m-column has been selected (HP-5MS UI 5%, 60 m × 250 μm, 0.25 μm, Agilent Technologies, USA) to allow for a proper separation of the total number of substances. Such a long analytical column results normally in a longer analysis time and potentially interfering peaks of the last eluting substances. Although the former effect was observed for the method studied here, the latter was not. The use of the selected column allowed the simultaneous analysis of such a large number of compounds in a single run with good resolution for the majority of substances.

Chromatographic analyses were performed in a GC equipped with a single quadrupole mass detector. The chromatographic column was supplied by Agilent Technologies Inc. (USA). All the GC-MS parameters are presented in Table 4.

**Table 4:** GC-MS instrumental parameters

Instrument	
Type	Gas chromatograph
Model	Agilent Technologies 7890 A
Column	
Stationary phase	HP-5MS UI 5% phenyl methyl siloxane
Dimensions	60 m × 250 μm, 0.25 μm
Flow rate	1.5 mL min^−1^
Carrier gas	Helium
Mode	Constant flow
Inlet	
Type	Split/splitless
Mode	Splitless
Inlet liner	Single taper liner
Temperature	300 °C
Purge on time	3 min
Purge flow	20 mL min^−1^
Oven	
Initial temperature	40 °C
Initial hold time	10 min
Ramp	6.75 °C min^−1^
Final temperature	315 °C
Final hold time	20 min
Run time	70.74 min
Detector	
Type	Agilent Technologies 5975C MSD
Operation mode	EI (Electron Impact); 70 eV
Mode	Total ion current (TIC) and extracted ion chromatogram (EIC)
Solvent delay	12 min
Injector	
Type	Automatic sampler
Injection volume	1 μL (10 μL syringe)

### Method performance

The proposed method was evaluated in terms of linearity, precision and trueness, limits of detection (LODs) and quantification (LOQs) according to method performance validation guidelines [21, 22]. The linearity was assessed by analysing standard solution mixtures at six concentration levels for each of the target analytes. The calibration curve was constructed with the ratio of the analyte peak area to the IS peak area. Two IS were used, namely 3-(4-isopropylphenyl)-2-methylpropionaldehyde and diphenyl phthalate. The former was employed for the quantification of the substances eluting up to its retention time (*t*_r_ = 32.17 min), hence the most volatile ones. Diphenyl phthalate was used as IS for all the remaining substances (*t*_r_ = 47.93 min). The linearity was evaluated by calculating the linear regression coefficient (*R*^2^). LODs and LOQs were evaluated from the chromatographic signal-to-noise ratio S/N. Mean value and standard deviation of the S/N were obtained from 5 chromatograms of blanks and the lowest calibration level for each substance, respectively. The LOD was estimated as analyte concentration providing an S/N of 3, while the LOQ was calculated as 3 times the LOD [21, 22].

Trueness and precision were assessed in food simulants A and C [2]. The respective food simulant was fortified at three concentration levels for all the selected analytes, based on their linear range, along with the IS. For short-term repeatability, six replicates of the fortified samples were analysed during the day, while for intermediate precision, six replicates of the aforementioned samples were analysed on three consecutive days. The trueness assessment of the analytical method was based on the calculation of the relative recovery as amount found in the fortified sample divided by the known amount added and expressed as percentage. The three tested concentrations for the short-term repeatability and intermediate precision have been selected either based on the SML [2] or on their LOQ [21, 22]. All results concerning trueness and precision are presented as Electronic Supplementary Material (ESM, Tables S1 to S4).

## Results and discussion

### Optimisation of the extraction from food simulant solutions

The main challenge was the selection of an organic solvent that could extract simultaneously and with good efficiency all the selected target FCM substances from the tested food simulants A and C.

Different organic solvents, namely hexane, isooctane, MTBE and DCM, were tested regarding the extraction efficiency for substances in simulant A, which is considered to be the “worst case” [2]. This efficiency was evaluated by comparing the amounts of each target analyte extracted from fortified food simulants (containing 250 ng mL^−1^) with the results obtained using their analytical standard solutions at the same concentration level. The results are presented in Table 5.

**Table 5:** LLE efficiency (%) of analytes at a concentration level of 250 ng/mL from food simulant A with different organic solvents

FCM no.	Target analyte	Extraction solvent				
		Hexane	Isooctane	MTBE	DCM (no salt)	DCM 10% NaCl
104	Hexadecyltrimethylammonium bromide	75.2	32.5	46.7	54.3	95.3
136	Camphor	98.5	124.2	99.5	96.9	92.1
138	Tri-n-butyl acetyl citrate	92.7	80.3	96.8	92.7	106.2
140	Triethyl citrate	70.2	0.0	0.0	70.2	109.0
142	Vinyltriethoxysilane	88.7	121.7	89.8	98.8	92.8
152	4,4′-Dichlorophenyl sulphone	91.8	97.8	90.8	91.8	102.5
153	Dapsone (4,4′-diaminodiphenyl sulphone)	0.00	0.0	0.0	95.5	109.6
155	α-Pinene	96.2	122.3	96.5	83.0	86.1
157	Dibutyl phthalate	92.7	105.2	107.3	91.9	108.2
159	Benzyl butyl phthalate	92.5	99.8	99.1	92.5	106.1
163	2,2′-Methylene bis(4-ethyl-6-tert-butylphenol)	89.7	99.2	105.1	97.5	110.1
171	Methyl benzoate	92.9	119.6	91.4	97.5	91.9
172	Ethyl benzoate	89.3	119.3	95.2	109.9	105.6
173	Propyl paraben	93.9	31.5	34.6	90.9	106.9
175	Allyl methacrylate	98.3	162.5	109.9	97.0	86.1
181	Ethyl methacrylate	92.9	119.8	94.2	83.5	82.1
183	Isobutyl methacrylate	98.5	126.8	99.3	94.2	89.8
184	Butyl methacrylate	85.5	14.6	21.5	85.5	107.4
185	Ethylene dimethacrylate	85.5	112.1	92.5	82.5	88.5
186	4-tert-Butylphenol	87.9	113.5	103.7	95.7	90.1
187	α-Methylstyrene	96.5	117.9	104.9	94.0	93.9
189	Methyl paraben	85.4	0.0	0.0	94.4	109.0
193	Styrene	100.1	138.1	104.0	91.6	86.4
195	Benzaldehyde	56.0	72.8	84.3	93.0	89.9
197	Cyclohexyl methacrylate	101.8	137.2	100.9	96.6	90.5
199	Resorcinol diglycidyl ether	111.9	189.9	145.6	112.9	119.1
206	2-Ethylhexyl acrylate	101.4	136.9	103.3	95.7	89.7
207	Bis(2-ethylhexyl) adipate	93.2	98.9	93.2	93.2	103.0
209	2-Ethyl-1-hexanol	95.6	93.7	94.8	93.1	91.6
212	Caprolactam	0.00	0.0	0.0	64.8	80.8
216	p-cresol	27.0	28.1	100.4	99.0	93.5
217	1,4-Dichlorobenzene	98.0	120.3	108.3	91.9	89.2
218	Isobutyl acrylate	86.8	119.1	87.9	96.7	90.8
220	Glycidyl methacrylate	64.4	90.1	94.5	97.8	84.8
241	Phenol	23.8	31.6	100.2	92.2	91.4
242	Dibutyl sebacate	84.5	98.2	111.7	84.5	105.4
271	Erucamide	92.4	90.6	98.6	92.4	107.1
283	DEHP	94.3	100.7	102.1	94.4	110.3
284	Methyl salicylate	88.7	124.4	106.7	99.3	92.1
285	2,2′-methylene bis(4-methyl-6-tert-butylphenol)	95.0	101.2	109.0	95.6	109.4
287	Ethyl paraben	86.6	0.0	0.0	88.5	105.4
288	Dimethyl terephthalate	86.9	86.4	90.2	86.9	105.7
293	Triethylphosphite as diethylphosphite (NIAS)	103.1	124.4	32.2	82.8	80.2
300	Butyl acetate	108.4	158.3	107.2	109.6	104.4
301	Butyl stearate	91.4	100.1	97.5	91.4	104.3
313	Diphenyl sulphone	91.7	23.8	28.2	91.7	100.8
314	β-Pinene	94.1	112.5	94.4	84.0	88.1
315	Butylated hydroxytoluene	91.4	83.1	100.2	87.4	104.1
316	Diallyl phthalate	86.2	95.6	98.9	86.2	102.1
318	2,4-Dihydroxybenzophenone	93.2	94.6	102.4	91.6	106.5
320	Butyl benzoate	99.7	113.1	101.6	95.3	99.3
322	Butyl lactate	53.8	65.7	96.3	100.2	92.9
325	n-Butyl acrylate	102.3	128.4	105.1	100.3	92.6
335	Oleamide	77.0	64.7	75.9	92.4	99.8
337	4,4′-Difluorobenzophenonen	89.4	87.8	101.8	89.4	103.5
342	Caprolactone	0.00	0.0	46.7	100.7	93.3
355	tert-Butyl methacrylate	99.0	163.1	104.0	96.0	84.4
371	Ethylene glycol monoacrylate	23.3	30.9	98.1	90.2	89.5
374	Ethylene glycol monomethacrylate	0.0	0.0	69.5	86.4	90.2
385	2-Hydroxypropyl acrylate	98.4	137.6	100.9	95.0	90.1
405	1,4-Divinyl benzene	94.3	132.5	100.1	98.9	93.1
405	1,2-Divinyl benzene	98.0	133.6	99.0	99.9	93.5
420	Dimethyl isophthalate	93.0	84.8	82.1	93.0	105.6
426	Bisphenol A glycidyl ether	97.0	158.8	196.1	95.1	117.8
431	2-Hydroxy-4-n-octyl benzophenone	102.8	92.9	123.3	99.8	108.7
433	Irganox 1076	106.7	96.3	114.1	106.7	107.3
434	1,4-Butanediol dimethacrylate	89.7	81.3	101.9	86.6	107.5
436	Vinyl laurate	85.6	88.3	99.7	85.6	104.7
437	Dodecyl acrylate	86.5	95.1	104.9	86.5	106.0
438	Bis (2,6-diisopropylphenyl)-carbodiimide	91.4	98.4	89.7	91.4	99.4
439	Phenyl methacrylate	102.8	134.1	103.0	98.8	93.1
441	Propyl benzoate	72.1	95.7	74.7	70.7	78.3
447	Benzyl methacrylate	89.8	92.7	97.3	89.8	102.4
453	Vinyltrimethoxysilane	90.7	117.2	55.6	62.8	78.7
457	sec-Butyl methacrylate	100.5	140.6	103.0	97.1	92.1
463	1,1,1-Trimethylolpropane trimethacrylate	90.2	96.4	102.0	88.2	106.4
487	Etocrilene	92.5	101.1	104.8	92.5	105.9
492	Octocrylene	99.9	106.6	132.6	97.9	113.3
497	2,2,4-Trimethyl-1,3-pentanediol diisobutyrate	91.8	95.0	87.1	91.8	105.0
671	Irgafos 168	97.3	90.7	96.2	97.3	105.5
788	3-Aminopropyltriethoxysilane	101.3	93.3	96.4	91.0	96.1
798	Dioctyl terephthalate	94.9	101.2	109.0	93.0	106.6
NIAS	(Z)-Dibutyl maleate	87.8	91.7	100.3	91.7	102.8
NIAS	Diisobutyl phthalate (DiBP)	92.7	98.8	99.3	93.6	102.4

It can be concluded that DCM with the presence of 10% w/v NaCl is the most suitable extraction solvent as it provided the best overall efficiency for a larger number of substances. Some of them could only be extracted with DCM, like ethylene glycol monomethacrylate (FCM No. 374), caprolactone (FCM No. 342), caprolactam (FCM No. 212) or dapsone (FCM No. 153). An extraction with isooctane presented subpar efficiency for a considerable number of substances. The addition of 10% m/v NaCl to DCM increased the extraction efficiency for many of the substances [20]. This effect was of particular importance for substances like caprolactam (FCM No. 212) and some parabens such as methyl paraben (FCM No. 189) and ethyl paraben (FCM No. 287). A notable example is hexadecyltrimethylammonium bromide (FCM No. 104), which showed low recoveries with MTBE and isooctane (< 48 %), an acceptable recovery with hexane (75 %) and up to 95% with DCM plus 10% NaCl. The effect of the salt addition during the LLE seems to be more significant with polar substances (such as phenol, FCM No. 241). However, a slight decrease in the DCM extraction efficiency was observed for substances with a lower polarity, where aprotic solvents are more efficient. Also, other substances, such as vinyltrimethoxysilane (FCM No. 453) or triethylphosphite (FCM No. 293), showed higher recoveries when using hexane and isooctane instead of DCM. In the end, a compromise had to be found and therefore DCM with the addition of 10% m/v NaCl has been selected because of the best overall results.

This extraction study was not performed under optimised precision and accuracy conditions because the objective at this stage was to screen and compare extraction efficiencies and not to validate the method. For instance, a complete baseline resolution was not obtained for certain substances and solvents, which has influenced the peak area calculation. Therefore, some of the reported extraction efficiencies are well above 100% in Table 5.

### Method validation

**Fig. 2:**
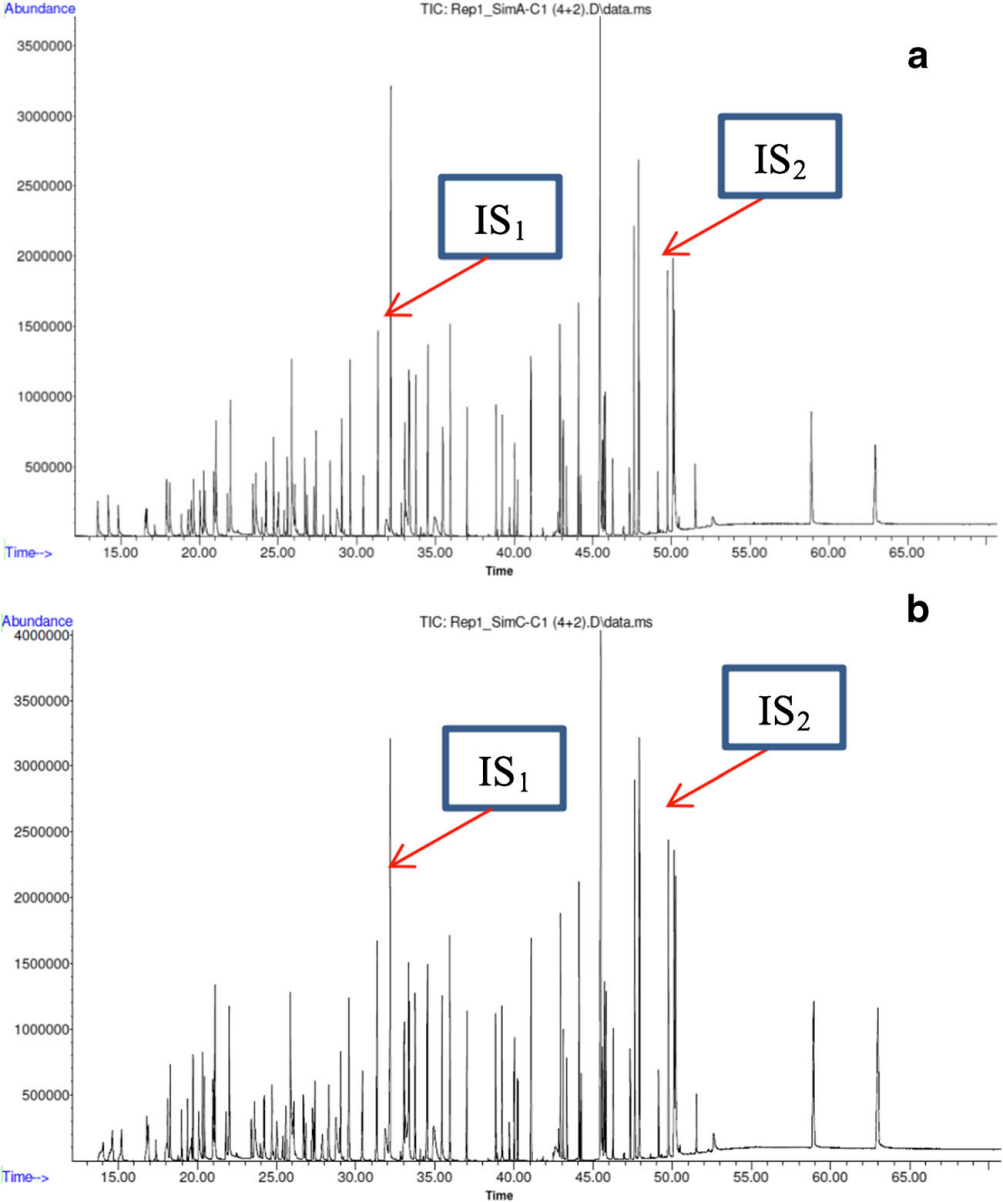
GC-MS total ion chromatograms of **a** fortified and extracted food simulant A at 2nd concentration level. **b** Fortified and extracted food simulant C at 2nd concentration level; IS 1: 3-(4-isopropylphenyl)-2-methylpropionaldehyde (0.5 μg mL^−1^); IS 2: diphenyl phthalate (0.5 μg mL^−1^)

In Fig. 2 are presented examples of the total ion chromatograms (TICs) of solutions resulting from the extraction of fortified food simulants with DCM + 10% NaCl.

The method was validated in-house and its LODs, LOQs and upper linear limits in food simulants A and C are presented in Table 6.

**Table 6:** Limits of detection (LOD), limits of quantification (LOQ) and upper linear limits for quantifying analytes in food simulants A and C

FCM no.	Target analyte	SML* (ng/ g^-1^)	Simulant A		Simulant C		Upper linear limit (ng mL^-1^)
			LOD (ng mL^-1^)	LOQ (ng mL^-1^)	LOD (ng mL^-1^)	LOQ (ng mL^-1^)	
104	Hexadecyltrimethylammonium bromide	6000.0	5.0	15.0	5.0	15.0	1250.0
136	Camphor	No	15.0	45.0	15.0	45.0	625.0
138	Tri-n-butyl acetyl citrate	60000.0	5.0	15.0	5.0	15.0	1250.0
140	Triethyl citrate	60000.0	8.0	25.0	8.0	25.0	1250.0
142	Vinyltriethoxysilane	50.0	15.0	45.0	15.0	45.0	625.0
152	4,4′-Dichlorophenyl sulphone	50.0	5.0	15.0	5.0	15.0	472.5
153	Dapsone (4,4′-diaminodiphenyl sulphone)	5000.0	15.0	45.0	20.0	60.0	1250.0
155	α-Pinene	No	25.0	75.0	25.0	75.0	625.0
157	Dibutyl phthalate	300.0	3.0	9.0	2.0	6.0	250.0
159	Benzyl butyl phthalate	30000.0	3.0	9.0	3.0	9.0	375.0
163	2,2′-Methylene bis(4-ethyl-6-tert-butylphenol)	1500.0	15.0	45.0	15.0	45.0	500.0
171	Methyl benzoate	No	8.0	24.0	8.0	24.0	625.0
172	Ethyl benzoate	No	5.0	15.0	8.0	25.0	375.0
173	Propyl paraben	No	8.0	25.0	8.0	25.0	1250.0
175	Allyl methacrylate	50.0	6.0	18.0	7.0	20.0	825.0
181	Ethyl methacrylate	6000.0	15.0	50.0	15.0	50.0	2500.0
183	Isobutyl methacrylate	6000.0	8.0	25.0	8.0	25.0	1250.0
184	Butyl methacrylate	6000.0	25.0	75.0	25.0	75.0	625.0
185	Ethylene dimethacrylate	50.0	6.0	15.0	6.0	15.0	1250.0
186	4-tert-Butylphenol	50.0	6.0	15	6.0	15.0	1000.0
187	α-Methylstyrene	50.0	6.0	18.0	6.0	18.0	1250.0
189	Methyl paraben	No	8.0	25.0	8.0	24.0	1250.0
193	Styrene	No	10.0	30.0	10.0	30.0	1250.0
195	Benzaldehyde	No	15.0	45.0	20.0	60.0	625.0
197	Cyclohexyl methacrylate	50.0	6.0	18.0	6.0	18.0	375.0
199	Resorcinol diglycidyl ether	ND**	40.0	120.0	40.0	120.0	1250.0
206	2-Ethylhexyl acrylate	50.0	6.0	18.0	6.00	18.0	375.0
207	Bis(2-ethylhexyl) adipate	18000.0	15.0	45.0	15.0	45.0	625.0
209	2-Ethyl-1-hexanol	30000.0	10.0	30.0	8.0	24.0	625.0
212	Caprolactam	15000.0	41.5	125.0	41.5	125.0	1250.0
216	p-Cresol	No	10.0	30.0	10.0	30.0	1250.0
217	1,4-Dichlorobenzene	12000.0	13.0	39.0	8.0	24.0	1250.0
218	Isobutyl acrylate	6000.0	12.5	40.0	12.5	40.0	625.0
220	Glycidyl methacrylate	20.0	4.0	12.5	4.0	12.5	1250.0
241	Phenol	No	8.0	24.0	8.0	24.0	625.0
242	Dibutyl sebacate	60000.0	20.0	60.0	25.0	75.0	1250.0
271	Erucamide	No	8.0	25.0	10.0	30.0	3750.0
283	DEHP	1500.0	3.0	9.0	3.0	9.0	1250.0
284	Methyl salicylate	30000.0	8.0	25.0	8.0	25.0	625.0
285	2,2′-Methylene bis(4-methyl-6-tert-butylphenol)	1500.0	15.0	45.0	15.0	45.0	500.0
287	Ethyl paraben	No	6.0	18.0	6.0	18.0	1250.0
288	Dimethyl terephthalate	No	5.0	15.0	5.0	15.0	625.0
293	Triethylphosphite as diethylphosphite (NIAS)	ND**	10.0	30.0	10.0	30.0	2500
300	Butyl acetate	No	20.0	60.0	20.0	60.0	1875.0
301	Butyl stearate	No	15.0	45.0	15.0	45.0	1250.0
313	Diphenyl sulphone	3000.0	3.0	9.0	3.0	9.0	625.0
314	β-Pinene	No	15.0	45.0	15.0	45.0	625.0
315	Butylated hydroxytoluene	3000.0	6.0	18.0	8.0	24.0	500.0
316	Diallyl phthalate	ND**	10.0	30.0	15.0	45.0	1250.0
318	2,4-Dihydroxybenzophenone	6000.0	33.3	100.0	40.0	120.0	1250.0
320	Butyl benzoate	No	4.0	12.0	4.0	12.0	375.0
322	Butyl lactate	No	20.0	60.0	25.0	60.0	2500.0
325	n-Butyl acrylate	6000.0	10.0	30.0	10.0	30.0	1250.0
335	Oleamide	No	13.0	40.0	20.0	60.0	2500.0
337	4,4′-Difluorobenzophenonen	50.0	5.0	15.0	5.0	15.0	625.0
342	Caprolactone	50.0	5.0	15.0	10.0	30.0	625.0
355	tert-Butyl methacrylate	6000.0	15.0	45.0	15.0	45.0	625.0
371	Ethylene glycol monoacrylate (2-hydroxyethyl prop-2-enoate)	6000	33.0	100.0	33.0	100.0	625
374	Ethylene glycol monomethacrylate	6000.0	15.0	45.0	15.0	45.0	3750.0
385	2-Hydroxypropyl acrylate	50.0	5.0	15.0	5.0	15.0	1250.0
405	1,4-Divinyl benzene	ND**	13.0	40.0	13.0	40.0	1250.0
	1,2-Divinyl benzene	-	15.0	45.0	15.0	45.0	1250.0
420	Dimethyl isophthalate	50.0	6.0	15.0	6.0	15.0	625.0
426	Bisphenol A glycidyl ether	No	10.0	30.0	30.0	90.0	1875.0
431	2-Hydroxy-4-n-octyl benzophenone	6000.0	13.0	40.0	10.0	30.0	2500.0
434	1,4-Butanediol dimethacrylate	50.0	6.0	15	6.0	18.0	1250.0
436	Vinyl laurate	No	15.0	45.0	15.0	45.0	1250.0
437	Dodecyl acrylate	50.00	6.0	15.0	6.0	15.0	1250.0
438	Bis (2,6-diisopropylphenyl)-carbodiimide	50.0	5.0	15.0	10.0	30.0	625.0
439	Phenyl methacrylate	6000.0	10.0	30.0	10.0	30.0	1250.0
441	Propyl benzoate	No	6.0	18	6.0	18.0	500.0
447	Benzyl methacrylate	6000.0	8.0	25.0	8.0	25.0	1250.0
453	Vinyltrimethoxysilane	50.0	15.0	50.0	15.0	50.0	1250.0
457	sec-Butyl methacrylate	6000.0	15.0	45.0	15.0	45.0	625.0
463	1,1,1-Trimethylolpropane trimethacrylate	50.0	4.0	12.0	4.0	12.0	1250.0
487	Etocrilene	50.00	5.0	15.0	10.0	30.0	375.0
492	Octocrylene	50.0	5.0	15.0	5.0	15.0	375.0
497	2,2,4-Trimethyl-1,3-pentanediol diisobutyrate	5000.0	5.0	15.0	5.0	15.0	1250.0
671	Irgafos 168	No	5.0	15.0	8.0	25.0	1250.0
788	[3-(methacryloxy)propyl]trimethoxysilane	50.0	5.0	15.0	5.0	16.0	1250.0
798	Dioctyl terephthalate	60000.0	5.0	15.0	5.0	15.0	1250.0
NIAS	(Z)-Dibutyl maleate	No	6.0	18.0	6.0	18.0	1250.0
NIAS	Diisobutyl phthalate	No	3.0	9.0	3.0	9.0	375.0

*Specific migration limit (mass of analyte per mass of food), according to Annex I, Table 1 of Reg. (EU) No. 10/2011[2]

**ND = the substance shall not migrate in detectable amounts

NA density of 1.0 g mL^−1^ was used as a factor for the mass fraction conversions; LODs and LOQs are expressed as analyte mass per volume of simulant.

For all the studied substances, the linear regression coefficients (*R*^2^) were higher than 0.99, indicating good linearity of the calibration curves. Regarding sensitivity, the followed guidelines [21, 22] and EU legislation requests that the LOQs should be at least 3 times lower than the SML of the substance. This was achieved for most of the substances in both food simulants A and C (Table 6). This requirement was even fulfilled for many of the substances with low SMLs (50.0 μg kg^−1^), like FCMs No. 175, 385, 187, 342, 197, 206, 186, 185, 788, 420, 434, 337, 437, 463, 487, 152, 438 and 492. The only two substances that could not be quantified at this SML were FCM No. 453 and 142, two silane-type substances. Also, FCM No. 220 (glycidyl methacrylate), with an SML of 20.0 ng g^−1^ and a method’s LOQ of 12.5 ng g^−1^ is challenging to be reliably quantified at its SML. For substance FCM No. 199, the required non-detection limit of 10 ng g^−1^ could not be reached. This was also the case for the individual substances forming FCM No. 405 for which the sum of divinylbenzenes and ethylvinylbenzenes should be non-detectable at a level of 10 ng g^−1^. For the substances without an SML, the existence of a proper analytical method as sensitive as possible is compulsory for their quantification in official food simulants A and C. The current method achieved low LOQs for all these substances. Overall, the LOQs for almost all substances are sufficient for their quantification at trace levels in official food simulants A and C [2, 21, 22].

The trueness and precision characteristics of the method were established with fortified food simulants A and C. All the results for the measurements in food simulant A are given in Tables S1 and S2 (see ESM), and the ones in food simulant C in Tables S3 and S4 (see ESM), respectively.

The results demonstrate the good precision of the method, with RSDs for the repeatability and intermediate precision below 15% for the determination of the majority of the substances. Some exceptions were observed for analytes belonging to the acrylates, namely FCM No. 206, 218, 355, 371 and 463. For these substances, RSDs were as high as 19.4% at some of the studied concentration levels. Recoveries were for the majority of substances between 70 and 115%.

### Analysis of real FCM samples

In order to evaluate the applicability of the method, 15 commercial FCM polymeric films were investigated. Migration tests were performed using different types of films, migration test conditions and food simulants according to their intended use (see Table 3 for migration test conditions). Results for the identified and quantified FCM substances are presented in Table 7.

**Table 7:** FCM regulated substances identified and quantified in the analysed polymeric film samples

Sample code	Sample type	Food simulant	FCM no. *	FCM substances*	Detected amounts (mg kg^−1^)
S 13	Multilayer	A	212	Caprolactam	2.74
			138	Tributyl acetyl citrate	0.17
			283	DEHP	0.07
			NIAS	DiBP	0.05
S 22	Multilayer	A	283	DEHP	0.04
			NIAS	DiBP	0.02
S 34	Monolayer	A	157	DBP	< 0.01
			283	DEHP	0.02
			NIAS	DiBP	0.07
S 41	Monolayer	A	209	2-Ethyl-1-hexanol	0.04
			157	DBP	0.01
S44	Monolayer	C	209	2-Ethyl-1-hexanol	0.21
			NIAS	DiBP	0.02
S 71	Monolayer	C	NIAS	DiBP	0.02

*According to Table 1 of Annex I of Reg. (EU) No. 10/2011 [2]

Eight out of the 14 commercial films, i.e. S5, S20, S25, S29, S31, S47, S49 and S62, did not release any of the 84 substances under the defined test conditions. The remaining six films released substances either below LOQ or up to 2.7 mg kg^−1^ food. The latter parameter has been calculated by taking into account the surface area which was in contact with the food simulant (A or C) in the migration test and a standard surface-to-food mass ratio of 6 dm^2^ kg^−1^ food that is prescribed for FCM films according to the Reg. (EU) No. 10/2011 [2]. The analysis of real FCM samples with food simulants A or C showed that the migrated amounts of the regulated substances of all tested materials were compliant with the requirements in the corresponding Regulation [2].

In addition to regulated substances, several NIAS were identified. Whether their migration is compliant with the Regulation depends on their quantification and risk assessment as reported by the producer.

### Experimental issues in the quantification of some FCM substances

During the method development, several observations with respect to the analysis of some of the substances have been made and are presented below.

During the quantification of triethylphosphite (FCM No. 293) in food simulants A and C, the presence of a second chromatographic peak was observed at a different retention time and with *m/z* of 82.0 Da, whereas this peak was not observed using a standard solution in organic solvents. This second peak was tentatively identified by using MS libraries as diethyl phosphite. This substance is not included in the positive list of the Reg. (EU) No. 10/2011; therefore, it is considered as a NIAS.

The presence of diethyl phosphite in aqueous food simulants could be a result of a hydrolysis, generating smaller alkyl phosphites or phosphorous acid [23, 24]. Another study using ^17^O-NMR investigated the hydrolysis of alkyl phosphites, including triethylphosphite, and observed the appearance of the di-alkyl product (Fig, 3). Since the Reg. (EU) No. 10/2011 specifies the use of aqueous food simulants, there is a priori a high probability that hydrolysis reactions would occur during the migration test. This could be especially the case during migration at high temperatures which could accelerate the hydrolysis rate [25, 26]. Therefore, the analysis of FCM No. 293 may become relatively difficult in aqueous simulants.

**Fig. 3:**
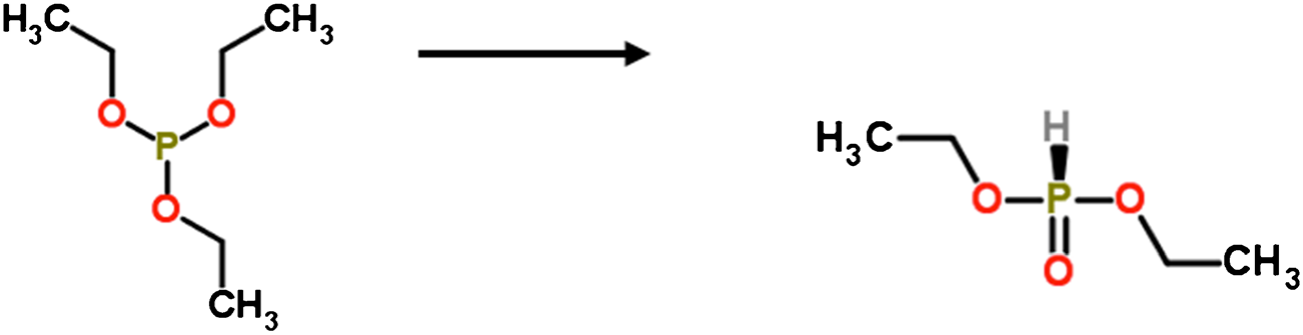
Potential hydrolysis of triethylphosphite (FCM No. 293) to diethylphosphite (NIAS)

Consequently, also diethyl phosphite has been included in the present multi-analyte method for being able to quantify FCM No. 293 indirectly. No hydrolysis of diethyl phosphite in fortified food simulants A and C was observed. The LOQ for the determination of diethyl phosphite was 25.0 ng mL^−1^ and accurate results were obtained in both food simulants A and C at the lowest concentration level studied. It is worth to note that according to the Reg. (EU) No. 10/2011, the verification of compliance of FCM products regarding FCM No. 293 is pending due to the unavailability of a proper analytical method. This may be related to the potential hydrolysis during the migration test in aqueous food simulants.

**Fig. 4:**
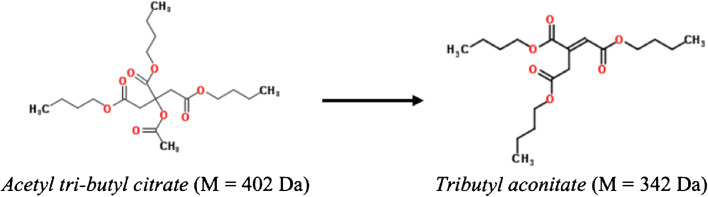
Hydrolysis of acetyl tributyl citrate (FCM No. 138) to tributyl aconitate (NIAS)

The hydrolysis of substance FCM No. 138, acetyl tributyl citrate, may also occur during the migration test in aqueous food simulants. This substance could be hydrolysed to tributyl aconitate. This was confirmed by studying a commercial standard and comparing its mass spectrum with those in MS libraries. The reaction product is also not listed in the Reg. (EU) No. 10/2011 and should be considered as a NIAS (Fig. 4). Although the hydrolysis rate seemed to be much lower than for FCM No. 293, it may have still affected the quantification.

## Conclusions

The multi-analyte method described here should support the efficient compliance control of FCM products regarding more than a few substances. The achieved method performance characteristics demonstrate that 84 substances in food simulants A and C can be analysed simultaneously. This number of analytes represents about 9% of the total number of authorised substances listed in the Regulation (EU) No. 10/2011.

## Electronic supplementary material

ESM 1 (PDF 214 kb).
