# Supplementary material for: Development and validation of a multi-analyte GC-MS method for the determination of 84 substances from plastic food contact materials
Source: Anal Bioanal Chem. 2020 Jun 24;412(22):5419–34. doi: 10.1007/s00216-020-02758-7 (PMC7387375; doi:10.1007/s00216-020-02758-7)
Supplement: Supplementary file 1 — (PDF 214 kb). [file 216_2020_2758_MOESM1_ESM.pdf]

Analytical and Bioanalytical Chemistry

Electronic Supplementary Material

Development and validation of a multi-analyte GC-MS method for the determination of 84 substances from plastic food contact materials

Emmanouil D. Tsochatzis, Joao Alberto Lopes, Eddo Hoekstra, Hendrik Emons

Table S1 Repeatability results for all the studied substances in food simulant A (10 % v/v aqueous ethanol)

FCM		1st Concentration level					2nd Concentration level					3rd Concentration level				
No	Substance	Added (ng mL^{-1})	Average found (ng mL^{-1})	SD* (ng mL^{-1})	RSD (%)	Recovery (%)	Added (ng mL^{-1})	Average found (ng mL^{-1})	SD * (ng mL^{-1})	RSD (%)	Recovery (%)	Added (ng mL^{-1})	Average found (ng mL^{-1})	SD * (ng mL^{-1})	RSD (%)	Recovery (%)
104	hexadecyltrimethylammonium bromide	**125**	91.6	0.2	0.2	73.3	**625**	499.9	70.5	14.1	80	**1250**	1248.8	2.1	0.2	99.9
136	Camphor	**62.5**	55.1	1.9	3.5	88.1	**312.5**	309.1	10.8	3.5	98.9	**625**	625.7	11.4	1.8	100.1
138	tri-n-butyl acetyl citrate	**125**	101.5	4.5	4.4	81.2	**625**	601.1	24.2	4	96.2	**1250**	1277.8	59.4	4.6	102.2
140	Triethyl citrate	**125**	103.8	3.8	3.6	83.1	**625**	504.3	8.3	1.6	80.7	**1250**	1226.5	49.9	4.1	98.1
142	Vinyl triethoxysilane	**62.5**	47.0	0.9	2.0	75.2	**312.5**	321.5	14.3	4.5	102.9	**625**	618	69.2	11.2	98.9
152	4,4'-dichlorophenyl sulfone	**47.25**	46.5	3.5	7.5	98.5	**236.3**	197.4	14.9	7.5	83.6	**472.5**	484.3	12.2	2.5	102.5
153	Dapsone (4,4'-diaminodiphenyl sulfone)	**125**	114.7	0.4	0.4	91.7	**0.6**	493.1	52.4	10.6	78.9	**1250**	1261.3	0.3	0	100.9
155	α-pinene	**62.5**	56.1	5.8	10.4	89.7	**312.5**	300.9	42.9	14.3	96.3	**625**	502.3	56.8	11.3	80.4
157	dibutyl phthalate	**25**	20.2	1.0	4.7	80.8	**125**	120.1	8.7	7.2	96.1	**250**	253.2	5.2	2	101.3
159	Benzyl Butyl phthalate	**37.5**	33.5	3.3	9.9	89.4	**187.5**	147.1	19.9	13.5	78.5	**375**	431.2	51.1	11.9	115
163	2,2'-methylene bis(4-ethyl-6-tert-butylphenol)	**50**	50.6	2.2	4.3	101.2	**250**	255	17.8	7	102	**500**	502.8	74.8	14.9	100.6
171	methyl benzoate	**62.5**	54.1	2.6	4.8	86.6	**312.5**	300.7	32.6	10.8	96.2	**625**	621.4	49.4	8	99.4
172	Ethyl benzoate	**37.5**	39.7	2.9	7.3	105.9	**187.5**	205.2	1.9	1.9	109.5	**375**	367.2	58.4	14.9	97.9
173	Propyl paraben	**125**	121	6.1	5.0	96.8	**625**	515.7	42.2	8.2	82.5	**1250**	1267.5	93.7	7.4	101.4
175	Allyl methacrylate	**82.5**	89.7	8.5	9.5	108.7	**412.5**	461.7	26	5.6	111.9	**825**	905.4	48.8	5.4	109.7
181	Ethyl methacrylate	**250**	228.5	29.8	13.1	91.4	**1250**	935.7	110.7	11.8	74.9	**2500**	2160.4	54.6	2.5	86.4
183	Isobutyl methacrylate	**125**	129.5	1.3	1.8	103.6	**625**	665.8	53.5	8	106.5	**1250**	1367.5	154	11.3	109.4
184	Butyl methacrylate	**62.5**	58.3	4.7	8.1	93.3	**312.5**	209.4	15	7.2	67	**625**	445.1	43.4	9.8	71.2
185	Ethylene dimethacrylate	**125**	129.7	9.9	7.6	103.8	**625**	601	52.2	8.7	96.2	**1250**	1214	81.9	6.7	97.1
186	4-tert butyl phenol	**100**	109.9	10.7	9.7	109.9	**500**	448	44.6	10	89.6	**1000**	1000.6	67	6.7	100.1
187	α-methyl styrene	**125**	104.3	2.4	2.3	83.4	**625**	636.7	6.7	1.1	101.9	**1250**	1248.7	222.9	17.9	99.9
189	methyl paraben	**125**	104.5	6.1	5.9	83.6	**625**	548.4	64.8	11.8	87.8	**1250**	1264.5	89.7	7.1	101.2
193	Styrene	**125**	102.3	1.7	1.6	81.8	**625**	590.2	24.4	4.1	94.4	**1250**	1247.7	213.9	17.1	99.8
195	Benzaldehyde	**62.5**	49.0	3.3	6.8	78.3	**312.5**	306.1	12.3	4	98	**625**	625.9	17.7	2.8	100.1
197	Cyclohexyl methacrylate	**37.5**	31.9	1.7	5.2	85.1	**187.5**	178.1	26.8	15	95	**375**	367.7	44.9	12.2	98.1
199	Resorcinol diglycidyl ether	**125**	141.6	8.2	5.8	113.2	**625**	516	49.1	9.5	82.6	**1250**	1401.1	20.8	1.5	112.1
206	2-ethylhexyl acrylate	**37.5**	35.4	0.5	1.5	94.4	**187.5**	152.8	6.3	4.1	81.5	**375**	363.4	62.5	17.2	96.9
207	bis(2-ethylhexyl) adipate	**62.5**	58.3	0.3	0.4	93.2	**312.5**	302.9	10.2	3.4	96.9	**625**	632.8	14.5	2.3	101.3
209	2-ethyl-1-hexanol	**62.5**	59.8	0.8	1.3	95.6	**312.5**	286.7	11.4	4	91.8	**625**	631	10.8	1.7	101

212	Caprolactam	**125**	113.9	9.9	8.7	91.1	**625**	670.2	68.2	10.2	107.2	**1250**	1331.2	38.7	2.9	106.5
216	p-cresol	**125**	107.3	1.5	1.4	85.8	**625**	597.6	37.5	6.3	95.6	**1250**	1255.8	41.8	3.3	100.5
217	1,4-dichlorobenzene	**125**	105.6	2.3	2.2	84.5	**625**	610.3	78	12.8	97.6	**1250**	1154.3	93.1	8.1	92.3
218	Isobutyl acrylate	**62.5**	47.6	4.3	9.1	76.1	**312.5**	331.5	60.5	18.3	106.1	**625**	633.4	6.3	1	101.3
220	Glycidyl methacrylate	**125**	104.5	0.7	0.6	83.6	**625**	608.7	29.4	4.8	97.4	**1250**	1249.4	29	2.3	100
241	Phenol	**62.5**	56.6	3.3	5.8	90.5	**312.5**	294.8	44.2	15	94.3	**625**	622.4	66.2	10.6	99.6
242	Dibutyl sebacate	**125**	106.8	11.9	11.1	85.4	**625**	586.5	46.1	7.9	93.8	**1250**	1268.7	147.3	11.6	101.5
271	Erucamide	**375**	319.7	4.2	1.3	85.3	**1875**	1612.1	23.2	1.4	86	**3750**	4368.6	78.8	1.8	116.5
283	DEHP	**125**	113.5	2.8	2.5	90.8	**625**	518.5	13.2	2.5	83	**1250**	1265.1	89.2	7.1	101.2
284	Methyl salicylate	**62.5**	55.3	5.5	10.0	88.4	**312.5**	305.2	48.2	15.1	97.7	**625**	632.6	76.5	12.1	101.2
285	2,2’-methylene bis(4-methyl-6-tert-butylphenol)	**50**	52.3	1.2	2.3	104.5	**250**	273.7	15.4	5.6	109.5	**500**	523.2	60.7	11.6	104.6
287	Ethyl paraben	**125**	105.3	1.5	1.4	84.3	**625**	445.4	29.8	6.7	71.3	**1250**	1263.2	138.2	10.9	101.1
288	dimethyl terephthalate	**62.5**	63.2	2.4	3.8	101.1	**312.5**	267.5	48	16	85.6	**625**	650.8	23.9	3.7	104.1
293	Triethhyl phosphite as **Diethyl phosphite (NIAS)**	**250**	233.7	25.0	10.7	93.5	**1250**	1187.9	90.7	7.6	95	**2500**	2476.4	317.8	12.8	99.1
300	Butyl acetate	**187.5**	146.6	19.8	13.5	78.2	**937.5**	976.4	34.7	3.6	104.2	**1875**	1918.4	83.2	4.3	102.3
301	Butyl stearate	**125**	118.8	3.7	3.1	95.1	**625**	542.7	11.4	2.1	86.8	**1250**	1261.5	75.6	6	100.9
313	Diphenyl sulfone	**62.5**	60.2	3.6	5.9	96.4	**312.5**	255.5	22.8	8.9	81.8	**625**	721.6	104.2	14.5	110.7
314	β-pinene	**62.5**	53.6	4.2	7.7	85.8	**312.5**	287.7	20.8	7.2	92.1	**625**	733.4	46	6.3	117.4
315	Butylated hudroxytoluene	**50**	48.8	1.1	2.2	97.6	**250**	241.9	19.1	7.9	96.8	**500**	489.6	31.3	6.4	97.9
316	Diallyl phthalate	**125**	118.7	1.6	1.3	95	**625**	579.3	34.1	6.1	92.7	**1250**	1265.4	91.4	7.2	101.2
318	2,4-dihydroxybenzophenone	**125**	98.0	6.4	6.6	78.4	**625**	609.6	71.1	11.7	97.5	**1250**	1213	129.4	10.7	97
320	butyl benzoate	**37.5**	26.5	0.2	0.7	70.6	**187.5**	199	22.4	11.3	106.1	**375**	393.5	34.2	8.7	104.9
322	Butyl lactate	**250**	209.4	11.7	5.6	83.8	**1250**	1227	2.6	0.2	98.2	**2500**	2486.1	12.1	0.5	99.5
325	n-butyl acrylate	**125**	123.6	8.7	7.1	98.8	**625**	546.7	25	4.6	87.5	**1250**	1357.2	71.7	6	108.6
335	Oleamide	**250**	202.4	0.9	0.5	81	**1250**	947	14.7	1.6	75.8	**2500**	2528.8	2.1	0.1	101.2
337	4,4'-difluorobenzophenonen	**62.5**	46.5	0.3	0.6	74.4	**312.5**	204.7	17.1	8.4	65.5	**625**	626	52.4	8.4	100.2
342	Caprolactone	**62.5**	55.6	2.0	3.5	89	**312.5**	303.8	24	7.9	97.2	**625**	621.3	34.5	5.6	99.4
355	tert-butyl methacrylate	**62.5**	62.9	2.9	4.6	100.7	**312.5**	310.4	50.5	16.3	99.3	**625**	630.9	51.1	8.1	100.9
371	Ethyleneglycol monoacrylate	**62.5**	70.0	5.1	7.2	112	**312.5**	267	47	17.6	85.4	**625**	533.9	113.1	21.2	85.4
374	Ethyleneglycol monomethacrylate	**375**	306.7	8.4	2.7	81.8	**1875**	1810.9	56.7	3.1	96.6	**3750**	3728.8	125	3.4	99.4
385	2-hydroxypropyl acrylate	**125**	110.1	2.4	2.2	88.1	**625**	586.3	20	3.4	93.8	**1250**	1267	23.9	1.9	101.4
405	divinyl benzene	**125**	125.2	23.3	18.6	100.1	**625**	673.2	25.7	3.8	107.7	**1250**	1332.8	6	0.5	106.6
420	dimethyl isophthalate	**62.5**	54.8	1.2	2.3	87.7	**312.5**	290.3	31.9	11	92.9	**625**	631.4	102.4	16.2	101
426	bisphenol A glycidyl ether	**187.5**	148.4	11.8	8.0	79.2	**937.5**	860.6	70.3	8.2	91.8	**1875**	1480.3	143.8	9.7	79

431	2-hydroxy-4-n-octyl benzophenone	**250**	209.9	1.0	0.5	84	**1.3**	1052.4	40.1	3.8	84.2	**2500**	2529.1	91.3	3.6	101.2
433	Irganox 1076	**375**	319.6	9.8	3.1	85.2	**1.9**	1784.6	204.5	11.5	95.2	**3750**	3826.9	363.2	9.5	102.1
434	1,4-butanediol dimethacrylate	**125**	102.5	16.2	15.1	82	**625**	534.6	14.7	2.7	85.5	**1250**	1292.3	142.6	11	103.4
436	Vinyl laurate	**125**	117.2	14.1	12	93.7	**625**	432.2	33.4	7.7	69.2	**1250**	1263.4	85.7	6.8	101.1
437	Dodecyl acrylate	**125**	117.6	1.3	1.1	94.1	**625**	553.7	28.8	5.2	88.6	**1250**	1463.5	49.1	3.4	117.1
438	bis (2,6-diisopropylphenyl)-carboiimide	**62.5**	67.2	0.2	0.4	107.5	**312.5**	303.8	27	8.9	97.2	**625**	633.1	75.7	12	101.3
439	phenyl methacrylate	**125**	98.2	0.1	0.1	78.6	**625**	648.9	43.5	6.7	103.8	**1250**	1258.3	34.9	2.8	100.7
441	Propyl benzoate	**50**	53.8	0.9	1.6	107.6	**250**	264.8	16.2	6.1	105.9	**500**	559.8	65.9	11.9	112
447	benzyl methacrylate	**125**	107.9	6	5.5	86.3	**625**	618.3	76.9	12.4	98.9	**1250**	1231.6	105.5	8.6	98.5
453	Vinyltrimethoxysilane	**125**	102.8	15.1	14.7	82.2	**625**	638	14.6	2.3	102.1	**1250**	1392.8	50.2	3.6	111.4
457	sec-Butyl methacrylate	**62.5**	56.0	6.5	11.5	89.5	**312.5**	312.5	51.4	16.4	100	**625**	445.1	50	11.2	71.2
463	1,1,1-trimethylolpropane trimethacrylate	**125**	106.7	6.2	5.8	85.4	**625**	563.3	67.1	11.9	90.1	**1250**	1428.3	93	6.5	114.3
487	Etocrilene	**37.5**	36.0	0.3	6.3	96	**187.5**	143.7	6.2	4.3	76.6	**375**	307.3	50.6	16.5	82
492	Octacrilene	**37.5**	40.2	0.8	4.9	107.2	**187.5**	149.6	20.1	20	79.8	**375**	407.8	54.8	13.4	108.7
497	2,2,4-trimethyl-1,3-pentanediol diisobutyrate	**125**	116	0.9	0.8	92.8	**625**	479.6	10.1	2.1	76.7	**1250**	1260.7	101.9	8.1	100.9
671	Irgafos 168	**125**	93.6	9.2	9.8	74.9	**625**	564.6	55.3	9.8	90.3	**1250**	1288.7	21.3	1.7	103.1
788	[3-(methacryloxy)propyl]-trimethoxysilane	**125**	91.8	1.4	1.5	73.4	**625**	612.8	69.7	11.4	98.1	**1250**	1235.1	51.6	4.2	98.8
798	di-octyl terephthalate	**125**	112.2	2.9	2.6	89.7	**625**	522	45.7	8.8	83.5	**1250**	1384.4	31.8	2.3	110.8
NIAS	(Z)-dibutyl maleate	**125**	110.9	0.6	0.6	88.7	**625**	547	29.3	5.4	87.5	**1250**	1280.9	94.9	7.4	102.5
NIAS	DiBP	**50**	51.9	7.0	13.5	103.7	**250**	242.9	18.1	7.5	97.2	**500**	514.3	26.4	5.1	102.9

* SD = Standard deviation

Table S2 Intermediate precision results for all the studied substances in food simulant A (10 % v/v aqueous ethanol)

FCM		1st Concentration level					2nd Concentration level					3rd Concentration level				
No	Substance	Added (ng mL^{-1})	Average Found (ng mL^{-1})	SD* (ng mL^{-1})	RSD (%)	Recovery (%)	Added (ng mL^{-1})	Average Found (ng mL^{-1})	SD* (ng mL^{-1})	RSD (%)	Recovery (%)	Added (ng mL^{-1})	Average Found (ng mL^{-1})	SD* (ng mL^{-1})	RSD (%)	Recovery (%)
104	hexadecyltrimethylammonium bromide	**125**	101.1	3.9	3.8	80.9	**625**	550.1	45.2	8.2	88	**1250**	1165.8	48.7	4.2	93.3
136	Camphor	**62.5**	57.9	1.8	3.2	92.7	**312.5**	306.4	9.1	3	98.1	**625**	615.8	14.4	2.3	98.5
138	tri-n-butyl acetyl citrate	**125**	105.1	6.6	6.3	84	**625**	541.5	13.1	2.4	86.6	**1250**	1198.7	53.1	4.4	95.9
140	Triethyl citrate	**125**	130.6	12.9	9.9	104.5	**625**	640	117.8	16.4	102.4	**1250**	1354.4	16.5	1.2	108.4
142	Vinyl triethoxysilane	**62.5**	57.2	0.7	1.3	91.5	**312.5**	328.3	7.5	2.3	105.1	**625**	669.6	46.8	7	107.1
152	4,4'-dichlorophenyl sulfone	**47.3**	46.2	2.1	4.6	97.9	**236.3**	174.1	17.7	10.2	73.7	**472.5**	472.2	63.4	13.4	99.9
153	Dapsone (4,4'-diaminodiphenyl sulfone)	**125**	117.8	1.7	1.4	94.2	**625**	469.9	26.9	5.7	75.2	**1250**	1034.7	6.6	0.6	82.8
155	α-pinene	**62.5**	54.1	3.7	6.8	86.6	**312.5**	338.5	26.1	7.7	108.3	**625**	634.6	59.9	9.4	101.5
157	dibutyl phthalate	**25**	28.3	1.6	5.7	113.2	**125**	122.8	6.6	5.4	98.2	**250**	228.5	10.3	4.5	91.4
159	Benzyl Butyl phthalate	**37.5**	36.2	2	5.5	96.4	**187.5**	141.1	12.3	8.7	75.2	**375**	368	38.6	10.5	98.1
163	2,2'-methylene bis(4-ethyl-6-tert-butylphenol)	**50**	52.6	1.1	2.1	105.2	**250**	185.6	4.3	2.3	74.2	**500**	408.5	49.7	12.2	81.7
171	methyl benzoate	**62.5**	59.7	3.6	6.1	95.6	**312.5**	293.4	17.9	6.1	93.9	**625**	596.1	31.7	5.3	95.4
172	Ethyl benzoate	**37.5**	38.8	2.7	6.9	103.5	**187.5**	155.7	9.1	5.8	83	**375**	308.2	8.9	2.9	82.2
173	Propyl paraben	**125**	126.6	11.5	9.1	101.3	**625**	504.9	15.9	3.1	80.8	**1250**	1116.6	76.5	6.9	89.3
175	Allyl methacrylate	**82.5**	87.7	8.5	9.7	106.3	**412.5**	461.7	26	5.6	111.9	**825**	905.4	48.8	5.4	109.7
181	Ethyl methacrylate	**250**	255.9	18.8	7.4	102.3	**1250**	1120.8	64.8	5.8	89.7	**2500**	2391.9	112.6	4.7	95.7
183	Isobutyl methacrylate	**125**	136.3	10.2	7.5	109.1	**625**	698.8	61.8	8.9	111.8	**1250**	1410.8	130.1	9.2	112.9
184	Butyl methacrylate	**62.5**	74.8	13.3	15.1	119.7	**312.5**	218.9	8.3	3.8	70.1	**625**	440.7	28.2	6.4	70.5
185	Ethylene dimethacrylate	**125**	97.4	9.5	9.8	77.9	**625**	575.1	30.6	5.3	92	**1250**	1150.3	56	4.9	92
186	4-tert butyl phenol	**100**	76.8	3.9	5.1	76.8	**500**	453.1	23.5	5.2	90.6	**1000**	984.3	46.8	4.8	98.4
187	?-methyl styrene	**125**	117	6.7	5.7	93.6	**625**	631.9	73.2	1.5	101.1	**1250**	1246.2	120.8	9.7	99.7
189	methyl paraben	**125**	89.3	7.6	8.6	71.5	**625**	497.1	46.4	9.3	79.5	**1250**	1197.5	87.9	7.3	95.8
193	Styrene	**125**	108.5	4.8	4.4	86.8	**625**	610.2	26.5	4.3	97.6	**1250**	1248.6	116.1	9.3	99.9
195	Benzaldehyde	**62.5**	46.6	6.7	14.4	74.5	**312.5**	299.8	8.8	3	96	**625**	618	19.1	3.1	98.9
197	Cyclohexyl methacrylate	**37.5**	32.4	1.2	3.8	86.4	**187.5**	162.3	4.6	2.8	86.6	**375**	344.1	25.4	7.4	91.8
199	Resorcinol diglycidyl ether	**125**	128	7.1	7.7	102.4	**625**	649	18.3	3.2	103.8	**1250**	1365.2	129.4	9.7	109.2
206	2-ethylhexyl acrylate	**37.5**	38.9	0.6	1.6	103.7	**187.5**	157	1.7	1.1	83.7	**375**	343.6	35	10.2	91.6
207	bis(2-ethylhexyl) adipate	**62.5**	66	0.3	0.5	105.6	**312.5**	311.5	7	3.1	99.7	**625**	555.3	26.2	4.7	88.8
209	2-ethyl-1-hexanol	**62.5**	59.8	0.8	1.3	95.6	**312.5**	286.7	11.4	4	91.8	**625**	631	10.8	1.7	101

212	Caprolactam	**125**	109.6	6.5	5.9	87.7	**625**	576.7	64.2	11.1	92.3	**1250**	1309	149.1	11.4	104.7
216	p-cresol	**125**	113	2.3	2	90.4	**625**	586.2	19	3.3	93.8	**1250**	1215.9	34.8	2.9	97.3
217	1,4-dichlorobenzene	**125**	129.2	3.5	2.7	103.3	**625**	691.5	41.6	6	110.6	**1250**	1281.5	134.3	10.6	102.5
218	Isobutyl acrylate	**62.5**	46.3	2.5	5.3	74	**312.5**	317.8	33.4	10.5	101.7	**625**	633	15.6	2.5	101.3
220	Glycidyl methacrylate	**125**	112.8	4.2	3.7	90.2	**625**	586.1	17.8	3	93.8	**1250**	1224.7	53.1	4.3	98
241	Phenol	**62.5**	63	4.2	6.7	100.9	**312.5**	284.8	22.2	7.8	91.2	**625**	574.4	42	7.3	91.9
242	Dibutyl sebacate	**125**	91.5	10.1	11	73.2	**625**	510.3	74.8	14.7	81.6	**1250**	1168.7	121.2	10.4	93.5
271	Erucamide	**375**	336.8	6.1	1.8	89.8	**1875**	1607.4	30.3	1.9	85.7	**3750**	3789.2	107.6	2.8	101
283	DEHP	**125**	122.3	1.8	1.5	97.8	**625**	482.7	30.6	6.3	77.2	**1250**	1104.1	80.7	7.3	88.3
284	Methyl salicylate	**62.5**	56.8	3.5	6.2	90.8	**312.5**	289.4	24.4	8.4	92.6	**625**	596.8	44.8	7.5	95.5
285	2,2'-methylene bis(4-methyl-6-tert-butylphenol)	**50**	51.1	0.9	1.8	102.3	**250**	183.9	8.4	4.5	73.6	**500**	432.8	46.5	10.7	86.6
287	Ethyl paraben	**125**	94.6	1.7	1.7	75.7	**625**	517	10.9	2.3	82.7	**1250**	987.5	80.2	8.1	79
288	dimethyl terephthalate	**62.5**	64	5.3	8.3	102.4	**312.5**	334.9	22.4	7	107.2	**625**	705.9	17.3	2.6	112.9
293	Triethhyl phosphite as **Diethyl phosphite (NIAS)**	**250**	267.8	24.9	9.3	107.1	**1250**	1032.7	73.8	7.2	82.6	**2500**	2159.3	190	8.8	86.4
300	Butyl acetate	**187.5**	176.4	9.9	5.6	94.1	**937.5**	982.1	22.3	2.3	104.8	**1875**	1912.9	88.2	4.6	102
301	Butyl stearate	**125**	125.9	2.9	2.3	100.7	**625**	443	25.9	5.8	70.9	**1250**	1088.6	71.4	6.6	87.1
313	Diphenyl sulfone	**62.5**	61.1	2.4	3.8	97.7	**312.5**	314.5	20.5	7	100.7	**625**	661.7	104.2	14.2	105.9
314	B-pinene	**62.5**	52.3	3.3	6.3	83.6	**312.5**	358.6	19.5	5.6	114.8	**625**	566.2	66.9	11.8	90.6
315	Butylated hudroxytoluene	**50**	49	0.9	1.8	98	**250**	181.6	22.3	12.3	72.6	**500**	440.4	26.1	5.9	88.1
316	Diallyl phthalate	**125**	122.6	1.6	1.3	98.1	**625**	502.8	19.8	3.9	80.4	**1250**	1190.1	132.3	11.1	95.2
318	2,4-dihydroxybenzophenone	**125**	100	8.1	8.1	80	**625**	658.2	68.5	10.4	105.3	**1250**	1141.4	73.6	6.5	91.3
320	butyl benzoate	**37.5**	31	0.6	1.9	82.5	**187.5**	164.3	12.3	7.5	87.6	**375**	297.7	19.8	6.7	79.4
322	Butyl lactate	**250**	237.4	7.1	3	94.9	**1250**	1121.4	1.7	0.2	89.7	**2500**	2270.2	48.5	2.1	90.8
325	n-butyl acrylate	**125**	107.6	12.6	11.7	86.1	**625**	628	54.9	8.7	100.5	**1250**	1218.1	100.5	8.3	97.5
335	Oleamide	**250**	206	1	0.5	82.4	**1250**	936.6	7.4	0.8	74.9	**2500**	2249.6	53.1	2.4	90
337	4,4'-difluorobenzophenonen	**62.5**	48.6	0.6	1.2	77.8	**312.5**	225.3	12.3	5.5	72.1	**625**	540.3	42.3	7.8	86.4
342	Caprolactone	**62.5**	58.8	1.8	3.1	94	**312.5**	299.3	13.1	4.4	95.8	**625**	603	25.7	4.3	96.5
355	tert-butyl methacrylate	**62.5**	63.5	4.5	7.1	101.6	**312.5**	323.9	27	8.4	103.6	**625**	668.6	47.2	7.1	107
371	Ethyleneglycol monoacrylate	**62.5**	51.1	5.7	11.1	81.8	**312.5**	237.9	41.8	17.6	76.1	**625**	613.1	129.9	21.2	98.1
374	Ethyleneglycol monomethacrylate	**375**	306.7	8.4	2.7	81.8	**1875**	1810.9	56.7	3.1	96.6	**3750**	3728.8	125	3.4	99.4
385	2-hydroxypropyl acrylate	**62.5**	75.7	1.8	2.3	121.1	**312.5**	368.2	49.5	13.4	117.8	**625**	768.2	78.2	7.1	122.9
405	divinyl benzene	**125**	142	5.2	3.7	113.6	**625**	688.2	56.1	8.2	110.1	**1250**	1329.3	25.3	2	106.3
420	dimethyl isophthalate	**62.5**	63.7	2.5	3.9	101.8	**312.5**	235.6	15.2	6.4	75.4	**625**	519	22.7	4.4	83
426	bisphenol A glycidyl ether	**187.5**	138.9	6	4.3	74.1	**937.5**	1051.3	146	13.9	112.1	**1875**	1862	197.3	10.6	99.3

431	2-hydroxy-4-n-octyl benzophenone	**250**	216.1	3.7	1.7	86.4	**1250**	1062	33.7	3.2	85	**2500**	2187.8	115.9	5.3	87.5
433	Irganox 1076	**375**	312.1	0.5	0.2	83.2	**1875**	1719.9	203.8	11.9	91.7	**3750**	3936.8	307.9	7.8	105
434	1,4-butanediol dimethacrylate	**125**	121.8	7.7	6.3	97.4	**625**	501.5	16.9	3.4	80.2	**1250**	1151.1	106.6	9.3	92.1
436	Vinyl laurate	**125**	120	16.8	14	96	**625**	514.7	20.3	4.4	82.4	**1250**	1083.1	69.6	6.4	86.7
437	Dodecyl acrylate	**125**	130.1	9.3	7.2	104.1	**625**	542.8	22.7	4.2	86.9	**1250**	1344.5	80	6	107.6
438	bis (2,6-diisopropylphenyl)-carboiimide	**62.5**	67.7	0.4	0.6	108.3	**312.5**	267.4	21.2	7.9	85.6	**625**	537	59.5	11.1	85.9
439	phenyl methacrylate	**125**	105.8	5	4.7	84.7	**625**	619.3	28.5	4.6	99.1	**1250**	1194.8	31.3	2.6	95.6
441	Propyl benzoate	**50**	46.8	5.8	12.5	93.6	**250**	265.7	50.1	19.3	106.3	**500**	580.5	90.5	15.6	116.1
447	benzyl methacrylate	**125**	112.1	4.5	4	89.7	**625**	600.1	40.1	6.7	96	**1250**	1198.6	67.8	5.7	95.9
453	Vinyltrimethoxysilane	**125**	104.7	18.2	17.4	83.7	**625**	591	8.2	1.4	94.6	**1250**	1179.4	148.3	12.6	94.4
457	sec-Butyl methacrylate	**62.5**	57.5	7.3	12.7	92	**312.5**	324.8	43.9	12.6	103.9	**625**	492	34.1	6.9	78.7
463	1,1,1-trimethylolpropane trimethacrylate	**125**	113	4.2	3.8	90.4	**625**	536	39.7	7.4	85.8	**1250**	951.3	565.1	19.4	76.1
487	Etocrilene	**37.5**	40.9	0.3	5.2	109.1	**187.5**	171.4	5	4.7	91.4	**375**	313	32.2	10	83.5
492	Octacrilene	**37.5**	34.2	1.4	7.4	91.2	**187.5**	159.6	16	10	85.1	**375**	417.8	68.9	14.5	111.4
497	2,2,4-trimethyl-1,3-pentanediol diisobutyrate	**125**	125.3	9	7.2	100.2	**625**	536.8	22.4	4.2	85.9	**1250**	1055.6	76.5	7.2	84.5
671	Irgafos 168	**125**	93.4	4.8	5.1	74.7	**625**	635.2	34.6	5.5	101.6	**1250**	1338.6	46.2	3.5	107.1
788	[3-methacryloxy)propyl] trimethoxysilane	**125**	98.4	3.5	3.5	78.7	**625**	594	35.7	6	95	**1250**	1203.6	37	3.1	96.3
798	di-octyl terephthalate	**125**	124.6	2.7	2.1	99.7	**625**	505.4	30.8	6.1	80.9	**1250**	1268.9	93.2	7.4	101.5
NIAS	(Z)-dibutyl maleate	**125**	116.7	4.1	3.5	93.3	**625**	538.3	35.3	6.6	86.1	**1250**	1176.2	83	7.1	94.1
NIAS	DiBP	**50**	51.2	6.9	13.5	102.4	**250**	240.0	17.9	7.5	96.0	**500**	508.0	26.1	5.1	101.6

* SD = Standard deviation

Table S3 Repeatability results for all the studied substances in food simulant C (20 % v/v aqueous ethanol)

FCM		1st Concentration level					2nd Concentration level					3rd Concentration level				
**No**	**Substance**	**Added**	**Average found**	**SD***	**RSD**	**Recovery**	**Added**	**Average found**	**SD***	**RSD**	**Recovery**	**Added**	**Average found**	**SD***	**RSD**	**Recovery**
		**(ng mL^{-1})**	**(ng mL^{-1})**	**(ng mL^{-1})**	**(%)**	**(%)**	**(ng mL^{-1})**	**(ng mL^{-1})**	**(ng mL^{-1})**	**(%)**	**(%)**	**(ng mL^{-1})**	**(ng mL^{-1})**	**(ng mL^{-1})**	**(%)**	**(%)**
104	hexadecyltrimethylammonium bromide	**125**	87.5	3	3.4	70	**625**	687.6	22.5	3.3	110	**1250**	1187.8	111.9	9.4	95
136	Camphor	**62.5**	61.2	4.4	7.2	98	**312.5**	312.4	16.2	5.2	100	**625**	626	19.3	3.1	100.2
138	tri-n-butyl acetyl citrate	**125**	123.7	7	5.6	99	**625**	603.4	56	9.3	96.5	**1250**	1277.7	55.1	4.3	102.2
140	Triethyl citrate	**125**	145.3	12	8.2	116.3	**625**	552	15.7	2.9	88.3	**1250**	1346.3	65.6	4.9	107.7
142	Vinyl triethoxysilane	**62.5**	66.9	5.4	8.1	107.1	**312.5**	313.4	29.5	9.4	100.3	**625**	631.5	10.3	1.6	101
152	4,4'-dichlorophenyl sulfone	**47.3**	48.2	5.1	10.5	102	**236.3**	241.2	10.3	4.3	102.1	**472.5**	472.1	68.6	14.5	99.9
153	Dapsone (4,4'-diaminodiphenyl sulfone)	**125**	113.1	4.6	4.1	90.4	**625**	638.7	2.1	0.3	102.2	**1250**	1247.9	136.5	10.9	99.8
155	α-pinene	**62.5**	68	1.8	2.7	108.9	**312.5**	351.6	48.9	13	112.5	**625**	630.1	45.1	7.2	100.8
157	dibutyl phthalate	**25**	21.8	0.9	3.9	87.4	**125**	116.8	19.1	15.4	93.4	**250**	249	39.1	15.7	99.6
159	Benzyl Butyl phthalate	**37.5**	37.3	3.6	9.5	99.4	**187.5**	178.8	22.9	12.8	95.4	**375**	376.7	47.5	12.6	100.4
163	2,2'-methylene bis(4-ethyl-6-tert-butylphenol)	**50**	40.8	1.2	3	81.5	**250**	235.5	34.8	14.8	94.2	**500**	508.4	73.2	14.4	101.7
171	methyl benzoate	**62.5**	58	6	10.3	92.9	**312.5**	307.9	42.4	13.8	98.5	**625**	616.1	68.7	11.2	98.6
172	Ethyl benzoate	**37.5**	34.5	0.2	0.6	92	**187.5**	185.9	6.1	3.3	99.2	**375**	370.3	10.7	2.9	98.7
173	Propyl paraben	**125**	128.9	2.8	2.2	103.1	**625**	539.7	29.1	5.4	86.4	**1250**	1305.3	52.2	4	104.4
175	Allyl methacrylate	**82.5**	78.6	9.5	12	95.3	**412.5**	437.8	58.6	11.6	106.1	**825**	837.7	57.3	6.8	101.5
181	Ethyl methacrylate	**250**	246.1	2.6	1.1	98.5	**1250**	1229.9	70.7	5.8	98.4	**2500**	2518.1	259.9	10.3	100.7
183	Isobutyl methacrylate	**125**	134	6.6	5	107.2	**625**	646.3	14.2	2.2	103.4	**1250**	1340.2	128.9	9.6	107.2
184	Butyl methacrylate	**62.5**	66.3	2.7	4.1	106.1	**312.5**	276.4	5.8	2.1	88.4	**625**	620.5	52.8	8.5	99.3
185	Ethylene dimethacrylate	**125**	112.1	1.2	1.1	89.7	**625**	624.7	49	7.9	100	**1250**	1224.8	102.6	8.4	98
186	4-tert butyl phenol	**100**	104.9	4	3.9	104.9	**500**	461	33.6	7.3	92.2	**1000**	1012.3	58.7	5.8	101.2
187	α-methyl styrene	**125**	115.5	6.8	5.8	92.4	**625**	537.1	29.9	5.6	85.9	**1250**	1275.5	82.1	6.4	102
189	methyl paraben	**125**	115	4.5	3.9	92	**625**	519.5	87.3	14.8	83.1	**1250**	1257.7	20.5	1.6	100.6
193	Styrene	**125**	112.3	13.3	11.9	89.8	**625**	635.9	34.9	5.5	101.7	**1250**	1232.4	53.2	4.3	98.6
195	Benzaldehyde	**62.5**	62.1	8.7	14	99.4	**312.5**	320.4	25.6	8	102.5	**625**	622.2	24.7	4	99.6
197	Cyclohexyl methacrylate	**37.5**	38.5	3.9	10	102.8	**187.5**	192.2	26.6	13.8	102.5	**375**	348.5	47.6	13.7	92.9
199	Resorcinol diglycidyl ether	**125**	126	9.3	7.3	100.8	**625**	634.2	85	13.4	101.5	**1250**	1263.7	94.4	7.5	101.1
206	2-ethylhexyl acrylate	**37.5**	35.7	1.5	4.1	95.2	**187.5**	156.3	10.2	6.5	83.3	**375**	323.9	27.7	8.6	86.4
207	bis(2-ethylhexyl) adipate	**62.5**	54.4	2.2	4.1	87	**312.5**	305.1	4.6	1.5	97.6	**625**	620.3	26.3	4.2	99.2
209	2-ethyl-1-hexanol	**62.5**	55.4	5.1	9.2	88.7	**312.5**	322.7	9.4	2.9	103.3	**625**	623.6	12.1	1.9	99.8

212	Caprolactam	**125**	124.1	5.8	4.7	99.3	**625**	650	34.1	5.2	104	**1250**	1150	80.4	7	92
216	p-cresol	**125**	106.1	6.7	6.3	84.9	**625**	629.9	27	4.3	100.8	**1250**	1200	163.9	13.7	96
217	1,4-dichlorobenzene	**125**	114	10.7	9.4	91.2	**625**	676.8	103.7	15.0	108.3	**1250**	1271.4	29.7	2.3	101.7
218	Isobutyl acrylate	**62.5**	58.5	7.9	13.5	93.6	**312.5**	343.5	16.8	5.1	109.9	**625**	711.2	24	3.4	113.8
220	Glycidyl methacrylate	**125**	106.1	6.5	6.1	84.9	**625**	635.3	38.8	6.1	101.7	**1250**	1245.7	31.7	2.6	99.7
241	Phenol	**62.5**	62	5.3	8.5	99.2	**312.5**	330.3	31.4	9.5	105.7	**625**	619.6	96.8	14.6	99.1
242	Dibutyl sebacate	**125**	127.3	1.8	1.4	101.9	**625**	605.2	16.1	2.7	96.8	**1250**	1268.7	47.6	3.8	101.5
271	Erucamide	**375**	318.2	33.2	10.4	84.8	**1875**	1927.3	76.7	4	102.8	**3750**	3760.3	135.9	3.6	100.3
283	DEHP	**125**	114.4	4.7	4.1	91.5	**625**	615.4	39.9	6.5	98.5	**1250**	1170.8	102.5	8.8	93.7
284	Methyl salicylate	**62.5**	62.9	2.7	4.4	100.7	**312.5**	307.5	51.8	14.9	98.4	**625**	576.4	26.1	4.5	92.2
285	2,2'-methylene bis(4-methyl-6-tert-butylphenol)	**50**	44.1	2.4	5.4	88.3	**250**	260.1	4.7	1.8	104.1	**500**	505.1	17.6	3.5	101
287	Ethyl paraben	**125**	110.5	2.4	2.2	88.4	**625**	576.3	20.5	3.6	92.2	**1250**	1228.7	19.6	1.6	98.3
288	dimethyl terephthalate	**62.5**	65.4	7.2	11	104.7	**312.5**	308.8	25.5	8.2	98.8	**625**	629.3	101.2	16.1	100.7
293	Triethhyl phosphite as **Diethyl phosphite (NIAS)**	**250**	243.6	7.1	2.9	97.5	**1250**	1153.3	130.3	11.3	92.3	**2500**	2736.5	338.4	12.4	109.5
300	Butyl acetate	**187.5**	178.6	6.5	3.7	95.2	**937.5**	867.4	133.5	15.4	92.5	**1875**	1892.2	116.5	6.2	100.9
301	Butyl stearate	**125**	87	0.9	2.1	69.6	**625**	410.8	15.5	3.8	65.7	**1250**	841	27.9	3.3	67.3
313	Diphenyl sulfone	**62.5**	63.3	5.7	9	101.4	**312.5**	294.9	37	12.6	94.4	**625**	669.7	17.4	2.6	107.1
314	a-pinene	**62.5**	55.1	3.8	6.9	88.1	**312.5**	244.4	31	12.7	78.2	**625**	606.6	69.8	11.5	97.1
315	Butylated hudroxytoluene	**50**	41.2	5.9	14.3	82.5	**250**	275.1	3.8	1.4	110	**500**	483.5	78.8	16.3	96.7
316	Diallyl phthalate	**125**	125	0.8	0.6	100	**625**	595	35.5	6	95.2	**1250**	1275.8	71.7	5.6	102.1
318	2,4-dihydroxybenzophenone	**125**	150.8	2.6	1.8	120.6	**625**	514.7	33.6	6.5	82.4	**1250**	1241.7	41.5	3.3	99.3
320	butyl benzoate	**37.5**	27.1	0.4	1.6	72.1	**187.5**	197.6	8.5	4.3	105.4	**375**	364.2	14.9	4.1	97.1
322	Butyl lactate	**250**	215.1	2.1	1	86.1	**1250**	1277	38.4	3	102.2	**2500**	2510.4	48.4	1.9	100.4
325	n-butyl acrylate	**125**	125.3	3.7	3	100.2	**625**	511.6	15.6	3.1	81.9	**1250**	1204.6	8.4	0.7	96.4
335	Oleamide	**250**	213.8	1	0.5	85.5	**1250**	1268.3	21.9	1.7	101.5	**2500**	2545	0.2	0	101.8
337	4,4'-difluorobenzophenonen	**62.5**	61.9	1.2	1.9	99.1	**312.5**	301.1	20.2	6.7	96.4	**625**	636.2	36.3	5.7	101.8
342	Caprolactone	**62.5**	60.1	5.2	8.6	96.1	**312.5**	311.3	25.9	8.3	99.6	**625**	619.5	45.1	7.3	99.1
355	tert-butyl methacrylate	**62.5**	65.7	1.7	2.6	105.1	**312.5**	335.9	7.8	2.3	107.5	**625**	642.7	98.1	15.3	102.8
371	Ethyleneglycol monoacrylate	**62.5**	59.7	2.4	4.1	95.5	**312.5**	288.3	27.2	9.4	92.2	**625**	724.7	68.8	9.5	116
374	Ethyleneglycol monomethacrylate	**375**	333.5	26.5	7.9	88.9	**1875**	1917.9	70.4	3.7	102.3	**3750**	3736.1	112	3	99.6
385	2-hydroxypropyl acrylate	**125**	113.9	11.2	9.8	91.1	**625**	612.5	19.7	3.2	98	**1250**	1261	51.6	4.1	100.9
405	divinyl benzene	**125**	120.8	5.5	4.5	96.6	**625**	643.1	71.4	11.1	102.9	**1250**	1272.7	41.6	3.3	101.8
420	dimethyl isophthalate	**62.5**	63.8	6.2	9.7	102	**312.5**	305.8	28.1	9.2	97.9	**625**	627.5	91.1	14.5	100.4
426	bisphenol A glycidyl ether	**187.5**	142.4	6	4.2	76	**937.5**	1048.1	24.2	2.3	111.8	**1875**	1834.8	121.7	6.6	97.9

431	2-hydroxy-4-n-octyl benzophenone	**250**	223.2	7.5	3.4	89.3	**1250**	1040.7	0.3	0	83.3	**2500**	2599	28.1	1.1	104
433	Irganox 1076	**375**	343.8	2.3	0.7	91.7	**1875**	1819.1	22.7	1.3	97	**3750**	3435.7	9.1	0.3	91.6
434	1,4-butanediol dimethacrylate	**125**	114.1	0.4	0.4	91.3	**625**	631.3	14.7	2.3	101	**1250**	1247.4	57.7	4.6	99.8
436	Vinyl laurate	**125**	117.2	2.8	2.4	93.8	**625**	649.9	29.3	4.5	104	**1250**	1254.3	32.4	2.6	100.4
437	Dodecyl acrylate	**125**	113.2	5.3	4.7	90.5	**625**	627	33.6	5.4	100.3	**1250**	1249.8	78.1	6.3	100
438	bis (2,6-diisopropylphenyl)-carboiimide	**62.5**	63.1	2.3	3.7	101	**312.5**	332.4	26.2	7.9	106.4	**625**	665.9	61	9.2	106.6
439	phenyl methacrylate	**125**	116.9	1	0.8	93.5	**625**	625	46.3	7.4	100	**1250**	1235.6	75.5	6.1	98.9
441	Propyl benzoate	**50**	40	2.2	5.6	80	**250**	259.7	10.4	4	103.9	**500**	455.6	32.2	7.1	91.1
447	benzyl methacrylate	**125**	126.2	4.2	3.3	101	**625**	615	75.4	12.3	98.4	**1250**	1234.1	136.4	11.1	98.7
453	Vinyltrimethoxysilane	**125**	131.3	13.8	10.5	105	**625**	595.9	93.7	15.7	95.4	**1250**	1256.7	4.6	0.4	100.5
457	sec-Butyl methacrylate	**62.5**	55.5	7.4	13.2	88.8	**312.5**	274.5	34.9	12.7	87.8	**625**	602.4	90.9	15.1	96.4
463	1,1,1-trimethylolpropane trimethacrylate	**125**	107.7	3.3	3	86.2	**625**	615.1	61.9	10.1	98.4	**1250**	1245.1	141.9	11.4	99.6
487	Etocrilene	**37.5**	42.6	3.3	7.8	113.6	**187.5**	197.7	1.1	0.6	105.5	**375**	348.8	22.5	6.4	93
492	Octacrilene	**37.5**	41.6	2.7	6.6	111	**187.5**	148	9.9	6.7	78.9	**375**	343.5	29.5	8.6	91.6
497	2,2,4-trimethyl-1,3-pentanediol diisobutyrate	**125**	123.5	6.7	5.4	98.8	**625**	621.8	59	9.5	99.5	**1250**	1242	107.3	8.6	99.4
671	Irgafos 168	**125**	90.6	1.4	1.6	72.5	**625**	598.9	44.4	7.4	95.8	**1250**	1242.5	87.4	7	99.4
788	[3-(methacryloxy)propyl]-trimethoxysilane	**125**	111.9	13.7	12.2	89.6	**625**	632.1	68.6	10.9	101.1	**1250**	1231.3	120.2	9.8	98.5
798	di-octyl terephthalate	**125**	114.1	1.4	1.2	91.3	**625**	621.6	36.7	5.9	99.5	**1250**	1247.2	80.9	6.5	99.8
NIAS	(Z)-dibutyl maleate	**125**	114	6.5	5.7	91.2	**625**	619.6	61.9	10	99.1	**1250**	1245.4	30.3	2.4	99.6
NIAS	DiBP	**50**	53.9	6.9	12.8	107.8	**250**	240.2	17.9	7.5	96.1	**500**	508.5	26.1	5.1	101.7

* SD = Standard deviation

Table S4 Intermediate precision results for all the studied substances in food simulant C (10 % v/v aqueous ethanol)

FCM		1st Concentration level					2nd Concentration level					3rd Concentration level				
No	Substance	Added (ng mL^{-1})	Average found (ng mL^{-1})	SD * (ng mL^{-1})	RSD (%)	Recovery (%)	Added (ng mL^{-1})	Average found (ng mL^{-1})	SD * (ng mL^{-1})	RSD (%)	Recovery (%)	Average found (ng mL^{-1})	Found (average)	SD * (ng mL^{-1})	RSD (%)	Recovery (%)
104	hexadecyltrimethylammonium bromide	**125**	101.2	4.6	4.6	81	625	672.4	14	2.1	107.6	**1250**	1155.2	111.9	9.7	92.4
136	Camphor	**62.5**	57.1	3.1	5.5	91.3	312.5	338	32.3	9.6	108.2	**625**	666.3	15.5	2.5	106.6
138	tri-n-butyl acetyl citrate	**125**	125	8.9	7.1	100	625	628.3	43.4	6.9	100.5	**1250**	1387.9	55.1	4	111
140	Triethyl citrate	**125**	130.5	15	10.8	104.4	625	697.6	33.7	4.8	111.6	**1250**	1431.5	65.6	4.6	114.5
142	Vinyl triethoxysilane	**62.5**	68.8	7.2	10.5	110	312.5	343.6	54.2	13.4	110	**625**	723.5	10.3	1. 6	115.
152	4,4'-dichlorophenyl sulfone	**47.3**	49.2	7	14.2	104.1	236.3	233	16.9	7.2	98.6	**472.5**	486.9	68.6	14.1	103
153	Dapsone (4,4'-diaminodiphenyl sulfone)	**125**	113.1	4.6	4.1	90.4	625	638.7	2.1	0.3	102.2	**1250**	1199.6	136.5	11.4	96
155	α-pinene	**62.5**	68.1	1.9	2.8	108.9	312.5	353.5	53.4	15.1	113.1	**625**	714.5	45.1	4.9	114.3
157	dibutyl phthalate	**25**	24.5	3	12.3	97.9	125	118.9	15.7	13.2	95.1	**250**	257.6	39.1	15.2	103.1
159	Benzyl Butyl phthalate	**37.5**	38	5.2	13.5	101.4	187.5	178	17.2	9.6	94.9	**375**	387.5	47.5	12.3	103.3
163	2,2'-methylene bis(4-ethyl-6-tert-butylphenol)	**50**	49.9	0.8	1.6	99.7	250	233.4	20.6	8.8	93.4	**500**	569.3	73.2	12.9	113.9
171	methyl benzoate	**62.5**	55.2	5.3	9.5	88.3	312.5	290.8	26.7	9.2	93	**625**	650.5	68.7	10.2	104.1
172	Ethyl benzoate	**37.5**	37	3.8	10.3	98.7	187.5	178.8	9.6	5.4	95.4	**375**	384.1	10.7	2.8	102.4
173	Propyl paraben	**125**	128.4	9.7	7.5	102.7	625	578.2	15.9	2.7	92.5	**1250**	1379	52.2	3.8	110
175	Allyl methacrylate	**82.5**	83.7	5.3	6.3	101.4	412.5	467.9	29.9	6.4	113.4	**825**	835.3	57.3	6.9	101.2
181	Ethyl methacrylate	**250**	263.4	5.9	2.2	105.4	1250	943.7	101.2	10.7	75.5	**2500**	2601.3	259.9	10	104.1
183	Isobutyl methacrylate	**0**	125.3	8.5	6.8	100.2	625	621.4	52	8.4	99.4	**1250**	1272.7	128.9	10.1	101.8
184	Butyl methacrylate	**62.5**	62.8	3.5	5.5	100.4	312.5	307.7	3.2	1	98.5	**625**	520.9	52.8	10.2	83.3
185	Ethylene dimethacrylate	**125**	113.3	3.9	3.4	90.6	625	578	52.5	9.1	92.5	**1250**	1149.7	102.6	8.9	92
186	4-tert butyl phenol	**100**	103.7	7.6	7.3	103.7	500	508.9	41.1	8.1	101.8	**1000**	1072.5	51.9	4.8	107.3
187	α-methyl styrene	**125**	113.7	3.4	3	90.9	625	537.1	29.9	5.6	85.9	**1250**	1123.6	82.1	7.3	89.9
189	methyl paraben	**125**	117.1	5.3	4.5	93.7	625	350.2	266.6	19	76	**1250**	1338.8	95.7	7.2	107.1
193	Styrene	**125**	109.3	10.9	10	87.4	625	651	52.5	8.1	104.2	**1250**	1210.8	53.2	4.4	96.9
195	Benzaldehyde	**62.5**	62.5	8.7	14	99.9	312.5	302.3	28	9.3	96.7	**625**	583.9	24.7	4.2	93.4
197	Cyclohexyl methacrylate	**37.5**	38.8	5	12.9	103.4	187.5	190.7	14.5	7.6	101.7	**375**	388.6	47.6	12.3	103.6
199	Resorcinol diglycidyl ether	**125**	123	10.1	8.2	98.4	625	561.7	50	8.9	89.9	**1250**	1299.3	94.4	7.3	104
206	2-ethylhexyl acrylate	**37.5**	41	4.7	11.6	109.2	187.5	180	15.7	8.7	96	**375**	385.1	38.1	9.9	102.7
207	bis(2-ethylhexyl) adipate	**62.5**	60.9	2.6	4.2	97.4	312.5	306.8	7.6	2.5	98.2	**625**	644.9	26.3	4.1	103.2
209	2-ethyl-1-hexanol	**62.5**	56.2	2.6	4.6	90	312.5	268.5	4.8	1.8	85.9	**625**	603.8	12.1	2	96.6

212	Caprolactam	**125**	116.4	5.9	5	93.1	625	666.5	44.5	6.7	106.6	**1250**	1095.5	399.9	2.7	87.6
216	p-cresol	**125**	112.8	5.5	4.9	90.2	625	593.6	44.9	7.6	95	**1250**	1057.9	163.9	15.5	84.6
217	1,4-dichlorobenzene	**125**	121.2	5.4	4.5	97	625	699.3	112.4	16.1	111.9	**1250**	1284	29.7	2.3	102.7
218	Isobutyl acrylate	**62.5**	59.4	6.9	11.6	95.1	312.5	342.4	28.3	8.5	109.6	**625**	664.5	24	3.9	106.3
220	Glycidyl methacrylate	**125**	116.4	9.7	8.3	93.1	625	662.3	60.2	9.1	106	**1250**	1308.5	31.7	2.4	104.7
241	Phenol	**62.5**	61.2	4.3	7	98	312.5	291.1	22.8	7.8	93.2	**625**	660.4	96.8	14.7	105.7
242	Dibutyl sebacate	**125**	122.1	1.8	1.5	97.7	625	615.2	24.4	4	98.4	**1250**	1261.5	48	3.8	100.9
271	Erucamide	**375**	327.3	17.7	5.4	87.3	1875	1902.7	83.9	4.4	101.5	**3750**	3918.6	135.9	3.5	104.5
283	DEHP	**125**	122.2	7.7	6.3	97.7	625	625.8	20	3.2	100.1	**1250**	1202.8	102.5	8.5	96.2
284	Methyl salicylate	**62.5**	65.2	5.4	8.2	104.3	312.5	320.4	27.6	8.6	102.5	**625**	652.3	26.1	4	104.4
285	2,2’-methylene bis(4-methyl-6-tert-butylphenol)	**50**	40.9	2.7	6.6	81.8	250	240.1	13.5	5.6	96	**500**	462.1	17.6	3.8	92.4
287	Ethyl paraben	**125**	122.8	5.3	4.3	98.2	625	589.4	27.4	4.6	94.3	**1250**	1205.6	19.6	1.6	96.5
288	dimethyl terephthalate	**62.5**	69.5	6.1	8.7	111.2	312.5	344.7	43.4	12.6	110.3	**625**	655.4	85.6	13.1	104.9
293	Triethhyl phosphite as **Diethyl phosphite (NIAS)**	**250**	271	5.9	2.2	108.4	**1250**	1352.2	123.3	9.1	108.2	**2500**	2547.3	338.4	13.3	101.9
300	Butyl acteate	187.5	172.9	5.8	3.4	92.2	937.5	991.4	67.6	6.8	105.8	1875	1963.1	116.5	6	104.7
301	Butyl stearate	**125**	96.9	6.1	6.5	77.5	625	452.6	11.7	2.8	72.4	**1250**	984.2	27.9	2.9	78.7
313	Diphenyl sulfone	**62.5**	64.7	7.4	11.5	103.5	312.5	297.8	28.3	9.5	95.3	**625**	670.5	17.4	2.6	107.3
314	β-pinene	**62.5**	58.9	3.9	6.6	94.2	312.5	313.5	39	12.5	100.3	**625**	621.2	69.8	11.2	99.4
315	Butylated hudroxytoluene	**50**	45.5	3.1	6.9	91	250	273.1	19.6	7.2	109.2	**500**	539	85	14.8	107.8
316	Diallyl phthalate	**125**	127.7	2.5	2	102.2	625	607.1	20.9	3.4	97.1	**1250**	1232.4	71.7	5.8	98.6
318	2,4-dihydroxybenzophenone	**125**	139.9	2.9	2.1	112	625	490.2	47.3	9.6	78.4	**1250**	1134.6	41.5	3.7	90.8
320	butyl benzoate	**37.5**	27.5	0.9	3.4	73.3	187.5	178.3	19.8	11.1	95.1	**375**	388.3	14.9	3.8	103.6
322	Butyl lactate	**250**	229.1	14.7	6.4	91.6	1250	1393.5	36	2.6	111.5	**2500**	2728.3	48.4	1.8	109.1
325	n-butyl acrylate	**125**	120.1	3.8	3.2	96.1	625	516	32.8	6.4	82.6	**1250**	1351.9	8.4	0.6	108.2
335	Oleamide	**250**	202.6	8.7	4.3	81	1250	1275.7	54.2	4.3	102.1	**2500**	2693.8	0.2	0	107.8
337	4,4'-difluorobenzophenonen	**62.5**	63.2	1.9	3	101.2	312.5	302.7	14.8	4.9	96.9	**625**	624.3	36.3	5.8	99.9
342	Caprolactone	**62.5**	55.9	4.4	7.9	89.4	312.5	336.6	30.4	9	107.7	**625**	652.5	69.6	7.8	104.4
355	tert-butyl methacrylate	**62.5**	63.6	4.7	7.3	101.8	312.5	334.8	14.9	4.5	107.2	**625**	667.2	98.1	14.7	106.8
371	Ethyleneglycol monoacrylate	**62.5**	59.4	2.4	4.1	95.1	312.5	289.9	30.5	10.5	92.8	**625**	698.8	68.8	9.8	111.8
374	Ethyleneglycol monomethacrylate	**375**	344.9	38.2	11.1	92	1875	1664.6	148.5	8.9	88.8	**3750**	3390.3	112	3.3	90.4
385	2-hydroxypropyl acrylate	**125**	113.8	11.2	9.8	91.1	625	614.9	24.7	4	98.4	**1250**	1240.3	51.6	4.2	99.2
405	divinyl benzene	**125**	119.2	3.1	2.6	95.4	625	682.8	42.6	6.3	109.2	**1250**	1350.3	41.6	3.2	108
420	dimethyl isophthalate	**62.5**	63.2	7.3	11.5	101.1	312.5	297.5	27.6	9.3	95.2	**625**	643.1	58.1	9	102.9
426	bisphenol A glycidyl ether	**187.5**	142.4	6	4.2	76	937.5	1048.1	24.2	2.3	111.8	**1875**	1791.7	121.7	6.8	95.6

431	2-hydroxy-4-n-octyl benzophenone	**250**	223.2	7.5	3.4	89.3	1250	1040.7	0.3	0	83.3	**2500**	2608.9	28.1	1.1	104.4
433	Irganox 1076	**375**	343.8	2.3	0.7	91.7	1875	1819.1	22.7	1.3	97	**3750**	3432.5	9.1	0.3	91.5
434	1,4-butanediol dimethacrylate	**125**	135.7	1.9	1.5	108.6	625	623.1	20	3.2	99.7	**1250**	1359.1	57.7	4.2	108.7
436	Vinyl laurate	**125**	120.4	4.2	3.5	96.3	625	613.7	17.8	2.9	98.2	**1250**	1288.4	74.5	5.8	103.1
437	Dodecyl acrylate	**125**	115.4	3.5	3	92.3	625	629.5	20.9	3.3	100.7	**1250**	1401	78.1	5.6	112.1
438	bis (2,6-diisopropylphenyl)-carboiimide	**62.5**	67.2	4.5	6.7	107.5	312.5	317.5	24	7.6	101.6	**625**	728.4	61	8.4	116.5
439	phenyl methacrylate	**125**	115.5	4.2	3.7	92.4	625	634.9	59.3	9.3	101.6	**1250**	1320.3	47.9	3.6	105.6
441	Propyl benzoate	**50**	42	4.9	11.7	83.9	250	256	6.8	2.7	102.4	**500**	505.2	31.5	6.2	101
447	benzyl methacrylate	**125**	128.8	6.3	4.9	103	625	616.3	62.7	10.2	98.6	**1250**	1193.9	136.4	11.4	95.5
453	Vinyltrimethoxysilane	**125**	130.2	12.6	9.7	104.2	625	634.5	71	8.3	101.5	**1250**	1349	4.6	0.3	107.9
457	sec-Butyl methacrylate	**62.5**	52.6	7.2	13.6	84.1	312.5	304.1	21.2	7	97.3	**625**	674.4	90.9	13.5	107.9
463	1,1,1-trimethylolpropane trimethacrylate	**125**	114.2	7.1	6.2	91.4	625	612.8	42.5	6.9	98	**1250**	1270.8	141.9	11.2	101.7
487	Etocrilene	**37.5**	37.9	4.3	8.3	101	187.5	188	10.6	5.6	100.3	**375**	372.2	22.5	6	99.3
492	Octacrilene	**37.5**	33.2	2	5.9	88.4	187.5	166	17.4	10.5	88.5	**375**	349	29.5	8.4	93.1
497	2,2,4-trimethyl-1,3-pentanediol diisobutyrate	**125**	123.4	10.8	8.8	98.7	625	606.7	35.1	5.8	97.1	**1250**	1354.4	107.3	7.9	108.4
671	Irgafos 168	**125**	90.6	1.4	1.6	72.5	625	598.9	44.4	7.4	95.8	**1250**	1211.6	87.4	7.2	96.9
788	[3-(methacryloxy)propyl]-trimethoxysilane	**125**	118.6	9.5	8	94.9	625	626.9	49.3	7.9	100.3	**1250**	1194.5	120.2	10.1	95.6
798	di-octyl terephthalate	**125**	123.7	7.6	6.1	99	625	617.6	23.6	3.8	98.8	**1250**	1336.8	80.9	6.1	106.9
NIAS	(Z)-dibutyl maleate	**125**	111.9	8.5	7.6	89.5	625	597	58.9	9.9	95.5	**1250**	1287.1	44.9	3.5	103
NIAS	DiBP	**50**	53.9	6.9	12.8	107.8	**250**	240.2	17.9	7.5	96.1	**500**	508.5	26.1	5.1	101.7

* SD = Standard deviation
